# Transcriptome analysis of resistant and susceptible Medicago truncatula genotypes in response to spring black stem and leaf spot disease

**DOI:** 10.1186/s12870-024-05444-3

**Published:** 2024-07-29

**Authors:** Jacob R. Botkin, Shaun J. Curtin

**Affiliations:** 1 Plant Science Research Unit, United States Department of Agriculture, St Paul, MN 55108 USA; 2 Department of Plant Pathology, University of Minnesota, St. Paul, MN 55108 USA; 3 Department of Agronomy and Plant Genetics, University of Minnesota, St. Paul, MN 55108 USA; 4 Center for Plant Precision Genomics, University of Minnesota, St. Paul, MN 55108 USA; 5 Center for Genome Engineering, University of Minnesota, St. Paul, MN 55108 USA

**Keywords:** RNA-seq, Host response, *Medicago truncatula*, Necrotrophic fungus, *Ascochyta medicaginicola*

## Abstract

Ascochyta blights cause yield losses in all major legume crops. Spring black stem (SBS) and leaf spot disease is a major foliar disease of *Medicago truncatula* and *Medicago sativa* (alfalfa) caused by the necrotrophic fungus *Ascochyta medicaginicola*. This present study sought to identify candidate genes for SBS disease resistance for future functional validation. We employed RNA-seq to profile the transcriptomes of a resistant (HM078) and susceptible (A17) genotype of *M. truncatula* at 24, 48, and 72 h post inoculation. Preliminary microscopic examination showed reduced pathogen growth on the resistant genotype. In total, 192 and 2,908 differentially expressed genes (DEGs) were observed in the resistant and susceptible genotype, respectively. Functional enrichment analysis revealed the susceptible genotype engaged in processes in the cell periphery and plasma membrane, as well as flavonoid biosynthesis whereas the resistant genotype utilized calcium ion binding, cell wall modifications, and external encapsulating structures. Candidate genes for disease resistance were selected based on the following criteria; among the top ten upregulated or downregulated genes in the resistant genotype, upregulated over time in the resistant genotype, hormone pathway genes, plant disease resistance genes, receptor-like kinases, contrasting expression profiles in QTL for disease resistance, and upregulated genes in enriched pathways. Overall, 22 candidate genes for SBS disease resistance were identified with support from the literature. These genes will be sources for future targeted mutagenesis and candidate gene validation potentially helping to improve disease resistance to this devastating foliar pathogen.

**Supplementary Information:**

The online version contains supplementary material available at 10.1186/s12870-024-05444-3.

## Introduction

Spring black stem and leaf spot (SBS) disease is a globally distributed disease of *Medicago truncatula* and *Medicago sativa* (alfalfa) [1]. Notably, SBS disease is one of the most severe foliar disease of alfalfa in Australia, Iran, Europe, and Canada [1–3]. The causal agent of SBS disease is *Ascochyta medicaginicola*, previously known as *Phoma medicaginis*. With the expansive genomic resources available for *M. truncatula*, this interaction presents an opportunity to study the host response to necrotrophic fungal pathogens of legumes [4]. The symptoms of SBS disease include necrotic lesions and chlorosis of the foliar tissue as well as the stems, which results in defoliation of the lower canopy. In alfalfa, yield losses are especially pronounced in the first or second harvest after a wet spring.

Complete resistance to SBS disease has not been observed. For resistant genotypes of *M. sativa* and *M. truncatula*, spore germination, penetration, and pycnidia development are delayed [2, 5]. Diseased plant material has higher amounts of the phytoestrogen coumestrol, which can impact livestock fertility [6]. South Australian Research and Development Institute (SARDI) maintains a large diverse collection of *M. truncatula*. Eighty-six of the SARDI *M. truncatula* accessions were screened for SBS disease response, and most were found to be susceptible; however, genotype-specific resistance was seen in 16 accessions, including SA27063, also known by the Medicago HapMap identifier HM078. On a 1 to 5 scale increasing in disease severity, HM078 has a mean disease rating of 1.64 against *A. medicaginicola* isolate OMT5, whereas the susceptible accession A17 (HM101) has a mean disease rating of 4.15 [2].

SBS-resistant accession SA27063 (HM078) and SBS-susceptible accessions A17 (HM101) and SA3054 were used as parents to generate two populations for quantitative trait locus (QTL) mapping that discovered *rnpm1* (HM101 & HM078) and *rnpm2* (SA3054 & HM078), two recessively inherited QTL which account for approximately 30% of the phenotypic variance for resistance to SBS disease of *M. truncatula* [7]. In addition, SA27063 (HM078) and SA3054 were also used as resistant and susceptible genotypes, respectively, in a microarray study of the host transcriptome at 12 h post inoculation (hpi) with *A. medicaginicola* [8]. In that study, Kamphuis et al. [8] found upregulation of the phenylpropanoid and octadecanoid pathways associated with defense responses. Another transcriptome study of SBS disease of alfalfa showed that several pathogenesis-related (PR) proteins were significantly upregulated upon infection with *A. medicaginicola* [9].

Plant defense responses to necrotrophic pathogens are complex and often differ from the host responses to biotrophic pathogens. There are two general arms of the plant immune system. First, an initial detection of pathogen associated molecular patterns (PAMPs) by transmembrane proteins called pattern recognition receptors (PRRs). PRR proteins are described as receptor-like kinases (RLKs) or receptor-like proteins (RLPs) that bind to PAMP ligands, and promote PAMP-triggered immunity (PTI). PTI includes callose deposition, lignification, an oxidative burst by reactive oxygen species (ROS), the production of PR proteins, the synthesis of antimicrobial compounds like phytoalexins, and production of plant hormones [10, 11]. Virulent pathogens possess effectors that dampen PTI. Plant disease resistance genes, also known as nucleotide-binding site and leucine-rich repeat (NLR) genes, function in sensor-helper pairs to detect effectors and initiate programmed cell death (PCD) [12]. The second arm of the plant immune system is the detection of these effectors by intracellular NLR proteins [13]. In the gene-for-gene model, NLR-mediated recognition of effector proteins results in effector-triggered immunity (ETI) and PCD. Specific NLR proteins have been shown to confer susceptibility to toxins of necrotrophic pathogens in the inverse gene-for-gene model also known as effector-triggered susceptibility (ETS) [14, 15]. Conversely, NLR proteins have also been found to confer resistance against necrotrophic fungi, such as the Dothideomycete pathogen *Leptosphaeria maculans* [16]. Resistance to necrotrophic pathogens has been associated with phytohormones such as salicylic acid (SA), jasmonic acid (JA), abscisic acid (ABA), and ethylene (ET), which regulate stress responses through signaling pathways [17]. For instance, the accumulation of JA in *Arabidopsis thaliana* has been associated with resistance to necrotrophic fungus *Sclerotinia sclerotiorum* [18]. Overall, plant immune responses need to be investigated in regard to specific pathosystems.

Comparative transcriptome analysis has been shown to be an effective method for identifying differentially expressed genes (DEGs) in response to plant-pathogen interactions. In this study, our objective was to identify candidate genes for SBS disease resistance for future validation in functional studies. We examined the host transcriptome of a resistant (HM078) and susceptible (A17) *M. truncatula* genotype at 24, 48, and 72 hpi with *A. medicaginicola*. We identified DEGs in the resistant and susceptible genotype compared to mock-treated samples at each time point and evaluated functionally enriched pathways. However, the number of DEGs was much lower in the resistant genotype. To identify candidate genes for disease resistance we examined the expression of SA and JA pathway genes, genes in QTL regions for disease resistance, RLKs, NLRs, and genes in functionally enriched pathways. We identified specific candidate genes based on five criteria; (1) among the top ten upregulated or downregulated genes in the resistant genotype, (2) upregulated DEGs over multiple time points in the resistant genotype, (3) DEGs in the susceptible genotype with higher constitutive expression in the resistant, (4) shared DEGs between resistant and susceptible with variable expression levels, or (5) genes in QTL regions *rnpm1* and *rnpm2* with contrasting expression profiles. We identified candidate genes for SBS disease resistance based on our comparative transcriptome analysis, functional annotations, and support from the literature. Overall, this study sheds light on the plant immune response to *A. medicaginicola* using contemporary genomic resources, and provides a number of strong candidate genes for SBS disease resistance to be validated in future studies.

## Methods

### Plant growth conditions

Germplasm of *M. truncatula* accessions A17 (HM101) and SA27063 (HM078) were obtained from the Medicago HapMap collection. Seed was scarified with 2 mL of concentrated sulfuric acid for 7 min, followed by washes with sterile de-ionized (DI) water. Seedlings were grown in autoclaved potting soil (Sun Gro Professional Growing Mix, Sun Gro Horticulture, Agawam, MA, USA) in a growth chamber at 22–24 °C with 16 h of light per day.

### Inoculation procedure

Fungal cultures were maintained on potato dextrose agar (PDA) and exposed to ambient daylight on the benchtop throughout growth. Inoculum of *A. medicaginicola* was prepared from 4-week-old cultures by flooding plates with 5 mL of sterile DI water with 50 ppm Tween^®^20 surfactant (Sigma-Aldrich, St. Louis, MO) and dislodging conidia into suspension. Conidial suspensions were strained using a Falcon™ Cell Strainer with a 40 μm pore (Thermo Fisher Scientific, Waltham, MA, USA) to remove hyphal fragments. Conidial suspensions were quantified using a hemocytometer under 400x magnification and adjusted to 5 × 10^5^ conidia/mL. The oldest trifoliate leaf originating from the node of the first secondary branch was marked with a white string tied to the petiole to be designated for inoculation. Approximately 1 mL of inoculum was atomized with a spray bottle at a distance of 15 cm away from the target leaf. Inoculated plants were placed in a humidity chamber at 100% relative humidity in the dark for 72 h following inoculation.

### Microscopic evaluation of SBS disease at selected time points

Spore germination and fungal growth on the leaf surface was observed for each genotype and time point. Cross sections of infected leaves were made to evaluate hyphal penetration. A sliding microtome was used to take 10 μm cross sections to visualize fungal penetration. The infected leaf material was immersed in GFP Polyclonal Antibody, Alexa Fluor^®^ 488 (496/518 nm) (Thermo Fisher Scientific, Waltham, MA, USA) in a phosphate buffered saline solution as previously described [19]. Alexa Fluor^®^ 488 selectively binds to N-acetylglucosamine, the monomer component of chitin, allowing for the fluorescence of hyphae under GFP (482/524 nm) wavelengths.

### RNA extraction and sequencing

At each time point (24, 48, and 72 hpi), three inoculated leaves and three mock-inoculated leaves were sampled from biological replicates of each genotype. A total of 36 inoculated leaves were harvested from SBS-resistant *M. truncatula* HM078 (*n* = 18) and SBS-susceptible A17 (*n* = 18) from 36 individual plants. Samples were not pooled between biological replicates. Leaves were harvested in low-light conditions. Tissue was stored at -80 °C until RNA extraction. Collected tissue was subjected to RNA extraction using the Qiagen RNeasy Mini Kit for Plants (Qiagen Inc., Valencia, CA, USA). For one sample an entire trifoliate leaf was homogenized in liquid nitrogen by mortar and pestle. Then, 75 mg of frozen tissue was sub-sampled, and added to 1 mL Buffer RLT (Qiagen). The rest of the protocol was followed according to the manufacturer’s specifications. Illumina RNA sequencing was conducted at the University of Minnesota-Twin Cities Genomics Center. TruSeq unique dual-indexed (UDI) stranded mRNA libraries were prepared, combined in a single pool, and sequenced on a single lane of NovaSeq S4 2 × 150-bp flow cell. Short-read RNA sequence data was uploaded to the Minnesota Supercomputing Institute for analysis.

### RNA sequence read alignment and quantification

RNA sequence reads derived from mock and inoculated plant tissue samples were processed in a series of steps detailed in the associated code file. First, Cutadadpt v1.18 [20] was used to trim Illumina sequencing adapters, retaining RNA sequence reads with Phred-scaled quality scores above 30, and reads longer than 50 bp. FastQC reports of RNA sequence data statistics were summarized with MulitQC v1.14 [21]. The *Mt5.0* reference genome of *M. truncatula* accession A17 was accessed from NCBI under RefSeq identifier GCF_003473485.1. The GFF file was converted to GTF format using the Cufflinks v2.2.1 function ‘gffread’ [22]. Next, STAR v2.5.3 [23] was used to perform spliced transcript alignments to the *Mt5.0* genome with the parameter ‘sjdbOverhang 149’ and the parameter ‘—sjdbGTFfile’ set to the *Mt5.0* GTF file. STAR v2.5.3 [23] was run in ‘twopassMode Basic’ and default parameters. The alignment data was quality checked using MulitQC v1.14 [21]. Samtools v1.9 [24] was used to merge and filter RNA sequence alignment files to include paired RNA sequence reads with unique alignments. For A17, 94.1% of reads aligned with a mean of 56.4 million reads per sample. For HM078, 91.3% of reads aligned with a mean of 55.9 million reads per sample (Additional file 2: Table S1). HTSeq-count v0.11.0 [25] was used to quantify the number of reads that mapped to each exon. Finally, a feature count matrix was generated showing the number of RNA sequence reads for each tissue sample that mapped uniquely to exons of each gene.

### Differential expression and pathway analysis

A count-based differential expression analysis was performed as previously described [26]. The EdgeR v3.36.0 pipeline [27] was used to conduct the differential expression analysis in R v4.1.2 [28]. First, a DGEList object was created from the feature count matrix and a model matrix object was created to store the experimental design variables for each sample. Next, the EdgeR v3.36.0 function ‘filterByExpr’ was used to determine which genes had adequate counts for statistical analysis across all samples. The function ‘calcNormFactors’ was used to determine the scaling factors to normalize counts based on library sizes, and the function ‘estimateDisp’ was used to evaluate common and tagwise dispersions. Then, the ‘glmQLFit’ function was used to fit the negative binomial generalized linear model (GLM) for each gene. Quasi-likelihood F-tests were performed for specified group comparisons using the ‘glmQLFTest’ function to calculate statistical differences in expression. For evaluating DEGs an absolute log_2_FC > 1 and an adjusted p-value (FDR) < 0.05 were used as cutoffs. A lower than standard log_2_FC cutoff was chosen due to relatively low numbers of DEGs in the resistant genotype. Statistical comparisons were made for each accession within each time point between mock and inoculated samples. DEGs were evaluated for enriched pathways using g:Profiler [29, 30]. Representative GO (Gene Ontology) terms were evaluated by Biological Processes (BP), Molecular Function (MF), Cellular Component (CC), and Kyoto Encyclopedia of Genes and Genomes (KEGG). Significantly enriched terms were retained if the adjusted p-value was below 0.05, or -log_10_(adjusted p-value) > 1.3.

### qPCR validation of RNA-seq gene expression data

To validate the RNA-seq results we performed quantitative RT-PCR (qPCR) on a set of eight genes. RNA was extracted using the Qiagen RNeasy Mini Kit for Plants (Qiagen Inc., Valencia, CA, USA), and synthesis of cDNA was performed using SuperScript IV Reverse Transcriptase (Thermo Fisher Scientific, Waltham, MA, USA). qPCR was performed using PerfeCta SYBR Green FastMix (Quanta BioSciences, Beverly Hills, California, USA) following the manufacturer’s recommendations. Amplification was performed for Nepenthesin (MtrunA17_Chr1g0187841), *MtKCS12* (MtrunA17_Chr2g0327721), *MtPP2C* (MtrunA17_Chr3g0105371), *MtEDS1L*-like (MtrunA17_Chr3g0118251), *MtCYP93C19* (MtrunA17_Chr4g0046331), *MtIFR* (MtrunA17_Chr5g0404481), *MtLAC7* (MtrunA17_Chr7g0240991), and *MtP21*-like (MtrunA17_Chr8g0386341). Primer pairs are detailed in Additional file 2: Table S2. qPCR was performed in triplicate for one biological replicate from each genotype at each time point, and mean C_t_ values for each sample were used for calculations. Relative quantification compared to the reference gene *MtACTIN11* (MtrunA17_Chr7g0223901) was calculated using 2^(-ΔΔC_T_) and (2^(-Δδlog_2_CPM)) for qPCR and RNA-seq data, respectively. Pearson’s correlation between fold change expression values was performed using R v4.1.2 in R studio v1.4.1717 [28].

## Results

### **Microscopic evaluation reveals invasive hyphae penetrating leaf tissue samples**

**Fig. 1:**
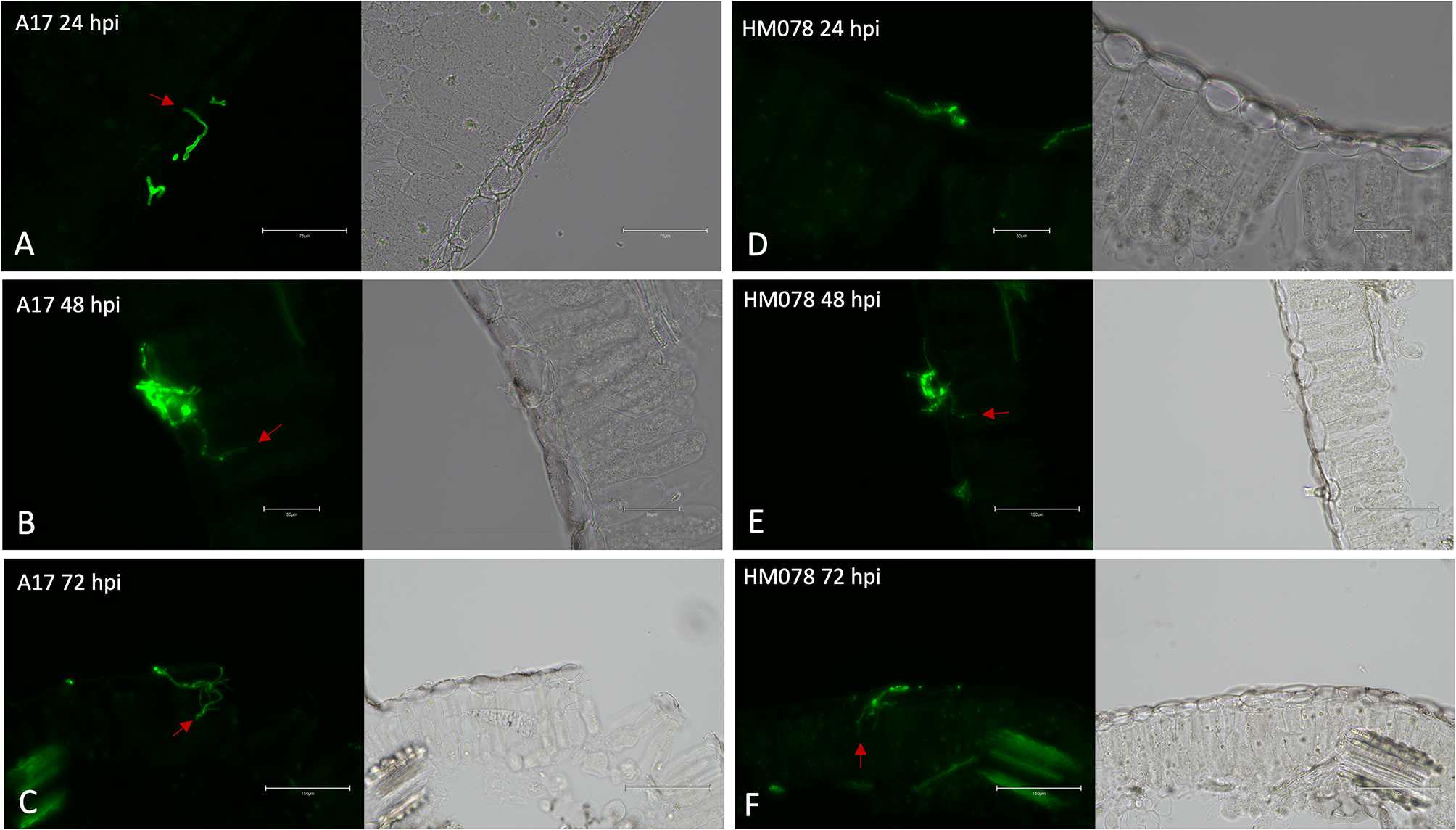
Cross sections of *M. truncatula* leaves infected with *A. medicaginicola*. Images were taken under GFP fluorescence (left) and RGB (right) for susceptible genotype A17 at (**A**) 24 hpi, (**B**) 48 hpi, and (**C**) 72 hpi, as well as the resistant genotype HM078 at (**D**) 24 hpi, (E) 48 hpi, and (**F**) 72 hpi. Red arrows indicate invasive hyphae penetrating leaf epidermal cells. Scale bars for (**A**-**F**) are 75, 50, 150, 50, 150, and 150 micrometers, respectively

To establish optimal harvest time points to capture the host-pathogen interaction, infected leaf cross sections of resistant (HM078) and susceptible (A17) *M. truncatula* genotypes were examined at 24, 48, and 72 hpi (Fig. 1). Invasive hyphae penetrating epidermal cells in cross sections of leaf samples were observed at each time point, except for the resistant genotype at 24 hpi. Notably, fewer invasive hyphae were observed on the resistant genotype in all samples along with reduced fungal growth on the leaf surface (Additional file 1: Figure S1). Overall, we concluded that the selected harvest time points were sufficient to capture the host response.

### Transcriptome analysis showed the largest differences in expression occurred at 72 hpi

Mock and inoculated *M. truncatula* leaf tissue of an SBS-resistant and SBS-susceptible genotype was collected at 24, 48, and 72 hpi for RNA sequencing. A total of 5.7 billion paired-end reads, with a mean library depth of 61.9 million reads per sample (Q > 30), were generated. After mapping reads to the *Mt5.0* reference genome, 25,084 genes had sufficient expression for statistical analysis across all time points. Across all samples, log_2_ counts per million (CPM) values ranged from − 4.87 to 15.54, with a mean of 1.95 log_2_CPM. Principal component analysis (PCA) of all samples revealed that genotype was the primary separating factor, followed by hpi (Additional file 1: Figure S2A). For both genotypes, PCA showed significant separation between mock and inoculated at 72 hpi. For the resistant genotype at 24 hpi, mock and inoculated samples overlap and show little variation, which may indicate transcription shifts due to pathogen inoculation were delayed (Additional file 1: Figure S2B). For the resistant genotype at 48 hpi, there was variability in mock and inoculated samples. For the susceptible genotype at 48 hpi, a single mock-inoculated sample appears to be an outlier, as it overlaps with the inoculated replicates (Additional file 1: Figure S2C).

### Differential expression analysis shows less response in the resistant genotype

**Fig. 2:**
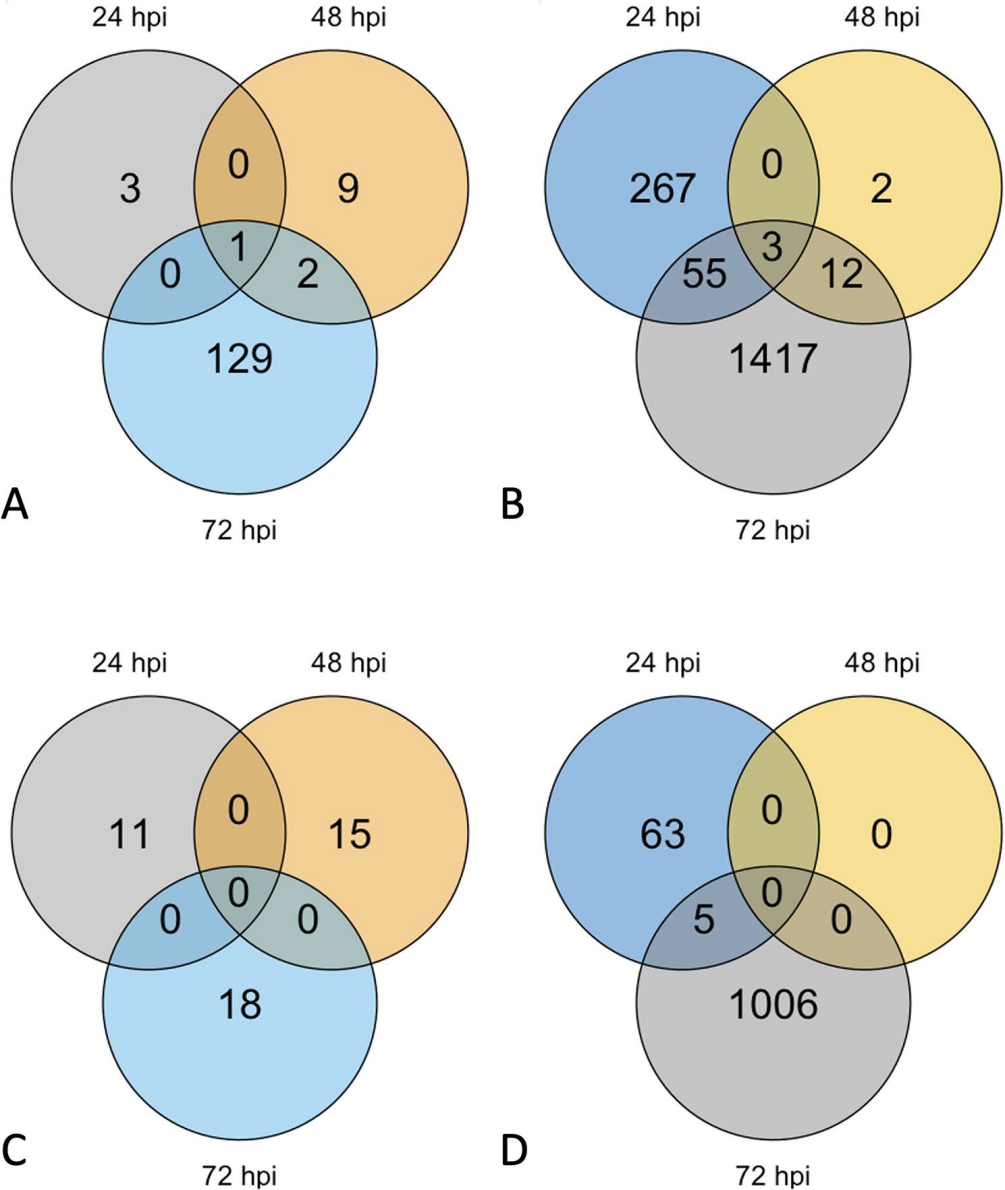
Number of DEGs for resistant and susceptible *M. truncatula* in response to *A*. ***medicaginicola***. Venn diagrams of (**A**) Upregulated DEGs of resistant genotype HM078, (**B**) Upregulated DEGs of susceptible genotype A17, (**C**) Downregulated DEGs of resistant genotype HM078, and (**D**) Downregulated DEGs of susceptible genotype A17

Differential gene expression from mock and inoculated, resistant and susceptible leaf tissue from three time points was analyzed (Fig. 2). Relevant statistics for the differential expression results at each time point are summarized for both host genotypes (Additional file 2: Table S3). Gene names and functional annotations are included when available. The resistant genotype HM078 had a total of 192 DEGs, including up and downregulation, which increased over time with 15, 27, and 150 DEGs at 24, 48, and 72 hpi, respectively (Additional file 1: Figure S3) (Additional file 2: Table S4). The susceptible genotype A17 had a total of 2,908 DEGs, including up and downregulation, with 393, 17, and 2,498 DEGs at 24, 48, and 72 hpi, respectively. The number of DEGs detected fits with observations made in the PCA. For instance, the high variability between biological replicates at 48 hpi likely contributed to low numbers of DEGs detected at this time point.

### Unique DEGs in the resistant genotype highlight potential genetic factors involved in disease resistance

The majority of the top ten most upregulated DEGs in the resistant genotype in response to pathogen infection were not differential expressed in the susceptible genotype. These included *MtPBP1*, *MtPrx28*, a MATH domain-containing protein, and a *MtRPP13*-like coiled-coil plant disease resistance protein (Table 1). Both calcium-binding protein *MtPBP1* and peroxidase *MtPrx28* are involved in ROS signaling and defense responses [31–34]. MATH domain-containing proteins have been shown to regulate NLR turnover in *A. thaliana* [35, 36]. The top ten most upregulated DEGs in the resistant genotype occurred at 72 hpi, while the top ten downregulated DEGs occurred at all time points. Notably, *MtPBP1* had a 600-fold increase in expression between mock and inoculated samples at 72 hpi. Overall, the identified genes are potentially strong candidates for SBS disease resistance.

**Table 1:** The ten most upregulated and downregulated DEGs in resistant genotype HM078

Time point (hpi)	Gene ID	Gene abbreviation	Function	log_2_FC	Unique DEG in HM078
72	MtrunA17_Chr2g0323211	*MtPBP1*	Putative calcium-binding protein KIC/PBP1/KRP1	9.23	Yes
72	MtrunA17_Chr4g0035371	N/A	Putative MATH domain-containing protein	8.89	Yes
72	MtrunA17_Chr8g0352621	*MtAnn2*	Putative annexin	7.66	Yes
72	MtrunA17_Chr2g0320061	*MtPrx28*	Putative peroxidase 28	7.49	Yes
72	MtrunA17_Chr7g0276531	N/A	Putative RNA-binding protein ARP1	7.10	No
72	MtrunA17_Chr8g0392521	*MtRPP13-like*	Putative disease resistance protein	6.25	Yes
72	MtrunA17_Chr5g0400461	N/A	Putative transcription factor NAM family	6.19	Yes
72	MtrunA17_Chr4g0006941	N/A	Putative non-specific serine/threonine protein kinase	6.01	Yes
72	MtrunA17_Chr1g0205731	N/A	Putative GTPase activating protein homolog 4	5.86	No
72	MtrunA17_Chr4g0042921	N/A	Putative transmembrane protein	5.81	Yes
24	MtrunA17_Chr0c27g0493921	*MtCTSH*	Putative Pro-cathepsin H	-6.85	Yes
24	MtrunA17_Chr3g0121991	*MtUGT75L6*	Putative crocetin glucosyltransferase	-5.76	No
48	MtrunA17_Chr8g0361011	N/A	Putative Ty3/gypsy retrotransposon protein	-5.01	Yes
24	MtrunA17_Chr4g0041901	*MtPPR*	Putative pentatricopeptide repeat-containing protein	-4.53	Yes
72	MtrunA17_Chr3g0081391	*MtRPM1-like*	Putative disease resistance protein	-4.24	Yes
48	MtrunA17_Chr1g0210081	N/A	Putative vacuolar protein sorting-associated protein Ist1	-3.43	Yes
48	MtrunA17_Chr4g0051271	N/A	Putative leucine-rich repeat domain superfamily	-3.31	Yes
24	MtrunA17_Chr7g0265101	N/A	Putative trans-zeatin O-beta-D-glucosyltransferase	-3.17	No
24	MtrunA17_Chr1g0196661	N/A	Putative glutathione transferase	-2.58	No
48	MtrunA17_Chr1g0188171	*MtCML10*	Putative Calmodulin-like 10	-2.43	No

Upregulated DEGs in the resistant genotype across multiple time points were identified, and the majority were not differentially expressed in the susceptible genotype (Table 2). These included the JA biosynthesis gene linoleate 9 S-lipoxygenase, *MtLOX1-5*, were upregulated across all three time points [37, 38]. A member of the 3-ketoacyl-CoA synthase family, *MtKCS12*, as well as a receptor-like kinase of the RLK-Pelle-DLSV family, were upregulated at 48 and 72 hpi. Genes in the 3-ketoacyl-CoA synthase family perform biosynthesis of very long chain fatty acids (VLCFA) [39, 40]. Transmembrane RLKs can act to recognize apoplastic pathogen effectors [41, 42]. No DEGs were shown to be downregulated in HM078 across multiple time points, although several were downregulated at 48 hpi and later upregulated at 72 hpi (Table 2). Overall, DEGs unique to the resistant genotype in response to pathogen infection highlight potential candidates for disease resistance.

**Table 2:** DEGs in the resistant genotype across multiple time points

Time point (hpi)	Gene ID	Gene abbreviation	Function	log_2_FC	Unique to HM078
48, 72	MtrunA17_Chr1g0210081	N/A	Putative vacuolar protein sorting-associated protein Ist1	-3.43, 3.04	Yes
48, 72	MtrunA17_Chr2g0299761	N/A	Putative nuclease HARBI1	-1.55, 1.68	Yes
48, 72	MtrunA17_Chr2g0327721	*MtKCS12*	Putative very-long-chain 3-oxoacyl-CoA synthase	3.32, 2.48	Yes
48, 72	MtrunA17_Chr4g0050141	N/A	Putative classical arabinogalactan protein	-1.98, 2.42	No
24, 48, 72	MtrunA17_Chr7g0272791	*MtLOX1-5*	Putative linoleate 9 S-lipoxygenase	1.72, 1.19, 1.09	Yes
48, 72	MtrunA17_Chr8g0345211	N/A	Putative pectinesterase	-1.29, 1.52	Yes
48, 72	MtrunA17_Chr8g0361341	N/A	Putative protein kinase RLK-Pelle-DLSV family	3.63, 3.23	Yes

Six of the top ten most downregulated DEGs in the resistant genotype were not differential expressed in the susceptible genotype during pathogen infection. These included genes like *MtCTSH*, *MtPPR*, and *MtRPM1-like* (Table 1). *MtCTSH*, a pro-cathepsin H, is a lysosomal cysteine protease, and while little is known about its role in plant disease, cathepsin B mediates PCD [43–45]. *MtPPR* is a pentatricopeptide repeat-containing protein, which are known to mediate post-transcriptional regulation [46]. In *A. thaliana*, the cleavage of a PPR protein has been associated with susceptibility to fungal disease, and these proteins share characteristics with plant disease resistance genes [47, 48]. *MtRPM1-like*, a coiled-coil plant disease resistance protein, was also downregulated in the resistant genotype. Interestingly, *RPM1* is required for resistance to *Pseudomonas syringae* in *Arabidopsis thaliana* and *Glycine max* [49, 50]. Overall, DEGs that are uniquely downregulated in the resistant genotype highlight potential genetic factors involved in the host response.

### Unique DEGs in the susceptible genotype provide insight into the compatible host response

When evaluating candidate genes for disease resistance from transcriptome data, it is beneficial to compare expression levels between contrasting host genotypes. Furthermore, analyzing DEGs in the susceptible genotype can provide insight into the compatible plant immune response to a necrotrophic fungus. In the susceptible genotype, the top ten most upregulated DEGs included *MtMYB* and *MtCHS-1 A* (Table 3). The MYB transcription factor family is involved in regulating a variety of stress responses, while chalcone synthases (*CHS*) are a vital component of flavonoid biosynthesis. The most downregulated DEG in the susceptible genotype was a major facilitator superfamily (MFS) transporter, which transport a variety of substrates across membranes (Table 3). Only three DEGs were upregulated across all time points in the susceptible genotype; cytochrome P450 monooxygenase *MtCYP76X2*, chalcone synthase *MtCHS-1 A*, and alcohol dehydrogenase *MtADH6.* Cytochrome P450s conduct NADPH or O_2_ dependent hydroxylation, while alcohol dehydrogenase oxidizes ethanol. An additional 67 DEGs were upregulated across multiple time points (Additional file 2: Table S5). Overall, the majority of DEGs in the susceptible genotype were not shared by the resistant genotype, and reveal a drastically different host response.

**Table 3:** The ten most upregulated and downregulated DEGs in susceptible genotype A17

Time point (hpi)	Gene ID	Gene abbreviation	Function	log_2_FC	Unique DEG in A17
72	MtrunA17_Chr1g0200151	N/A	putative protein	10.97	Yes
72	MtrunA17_Chr4g0024181	*MtMYB*	Putative transcription factor MYB-HB-like family	10.81	Yes
72	MtrunA17_Chr5g0400961	N/A	Putative VQ motif-containing protein	10.72	Yes
72	MtrunA17_Chr7g0266071	*MtLYE1*	LysM domain containing protein	10.55	Yes
72	MtrunA17_Chr3g0121541	*MtCHS-1 A*	Chalcone synthase 1 A	10.34	Yes
72	MtrunA17_Chr5g0414361	N/A	putative protein	10.31	Yes
72	MtrunA17_Chr7g0218261	N/A	Putative methyltransferase	10.26	Yes
72	MtrunA17_Chr4g0051421	N/A	Putative tetrahydroberberine oxidase	10.21	Yes
72	MtrunA17_Chr4g0048041	N/A	Putative NADH: ubiquinone reductase (non-electrogenic)	10.05	Yes
72	MtrunA17_Chr7g0218841	N/A	Putative UDP-glucuronosyl/UDP-glucosyltransferase	10.04	Yes
72	MtrunA17_Chr3g0111301	N/A	hypothetical protein	-6.26	Yes
72	MtrunA17_Chr6g0467861	N/A	Putative shikimate O-hydroxycinnamoyltransferase	-6.43	Yes
72	MtrunA17_Chr3g0108631	N/A	Putative NAD(P)H-quinone oxidoreductase, subunit N	-6.43	Yes
72	MtrunA17_Chr5g0444611	N/A	putative protein	-6.49	Yes
72	MtrunA17_Chr4g0026681	N/A	putative protein	-6.50	Yes
72	MtrunA17_Chr8g0354041	*MtEIX2-like*	Putative non-specific serine/threonine protein kinase	-6.58	Yes
72	MtrunA17_Chr3g0142421	N/A	Putative small auxin-up RNA	-6.86	Yes
72	MtrunA17_Chr3g0124411	N/A	Putative heavy metal binding protein HIPP/ATX1	-6.87	Yes
72	MtrunA17_Chr6g0486091	N/A	Putative adenosylhomocysteine nucleosidase	-7.00	Yes
72	MtrunA17_Chr3g0143461	N/A	Putative MFS transporter superfamily	-7.43	Yes

### Shared DEGs by both host genotypes include a variety of transcription factors

A total of 65 genes were differentially expressed in both the resistant and susceptible genotypes (Additional file 2: Table S6). The top upregulated DEG in both genotypes was RNA-binding protein ARP1. A variety of transcription factor gene families were among the shared DEGs, which included C2H2, AS2-LOB, Calmodulin-binding, Homeobox-WOX, WD40, WRKY, C2C2-Dof, and AP2-EREBP. Ten TFs were differentially expressed in both genotypes; seven were upregulated and two were downregulated. A WD40-type strictosidine synthase-like 10 transcription factor, *MtSTR10-*like, was downregulated in the susceptible genotype while upregulated in the resistant genotype. Strictosidine synthases have been implicated in terpenoid biosynthesis of phytoalexins [51, 52]. The resistant genotype showed higher upregulation for six of the seven upregulated transcription factors. For example, the C2H2 transcription factor *MtZAT11* had a 36-fold increase in the resistant genotype, but only a 6-fold increase in the susceptible genotype at 72 hpi. C2H2 transcription factors have been shown to be involved in regulation of hormone pathways in response to biotic stress [53, 54]. Finally, for the two downregulated TFs, the resistant genotype showed less downregulation.

### Functional enrichment analysis provides insight into contrasting host responses

**Fig. 3:**
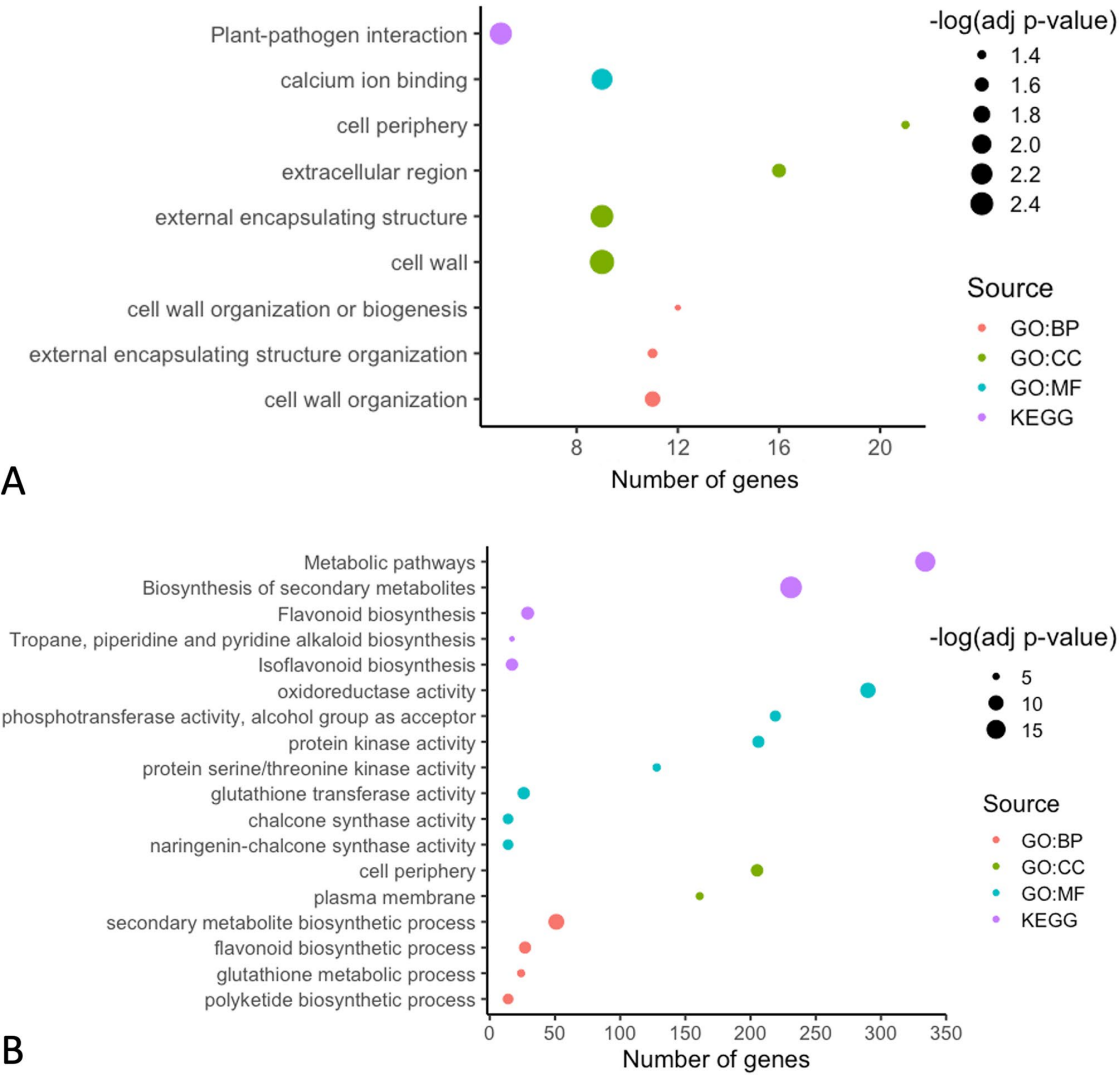
Functional enrichment analysis of resistant and susceptible *M. truncatula* in response to *A. medicaginicola*. Significantly enriched GO terms were analyzed for (**A**) DEGs in the resistant genotype HM078, and (**B**) DEGs in the susceptible genotype A17. Upregulated and downregulated DEGs across all time points were included for each genotype. GO (Gene Ontology) terms were grouped by Biological Processes (BP), Molecular Function (MF), Cellular Component (CC), or Kyoto Encyclopedia of Genes and Genomes (KEGG) pathways

Functional enrichment analysis of DEGs revealed that the resistant and susceptible genotypes activate distinct pathways. We examined upregulated and downregulated DEGs across all time points for each genotype, and reported significantly enriched terms (Fig. 3). In the resistant genotype, the most significant cellular components included ‘cell wall’, ‘external encapsulating structure’, and ‘extracellular region’ (Fig. 3A). The most significant biological processes were ‘cell wall organization’ and ‘external encapsulating structure or organization’. The most significant molecular function was ‘calcium ion binding’. Overall, due to fewer DEGs, the functional enrichment analysis in the resistant genotype was limited. For instance, KEGG enrichment analysis resulted in one significantly term, ‘Plant-pathogen interaction’.

For the susceptible genotype, the most significant cellular components were ‘cell periphery’ and ’plasma membrane’ (Fig. 3B). The most significant biological processes were ‘secondary metabolite biosynthetic process’ and ‘flavonoid biosynthetic process’. The most significant molecular function was ‘oxidoreductase activity’. KEGG enrichment analysis showed engagement in ‘Biosynthesis of secondary metabolites’, ‘Metabolic pathways’, and ‘Flavonoid biosynthesis’.

### Regulation of hormone pathways in response to *A. Medicaginicola*

The SA and JA pathways are crucial for plant immune responses. A previous study has shown that *M. truncatula* activates these pathways in response to *A. medicaginicola* [8]. In the compatible interaction with the susceptible genotype, there is a greater induction in the SA pathways, whereas the resistant genotype shows a rapid induction of the JA pathway [8]. Expression profiles for genes contributing to SA and JA biosynthesis and signaling were visualized in a heatmap (Additional file 1: Figure S4). Across all three time points, the resistant genotype upregulated the JA biosynthesis gene *MtLOX1-5* (Table 4). *MtLOX1-5* was highlighted earlier for being one of a few DEGs upregulated in the resistant genotype over multiple time points, although the susceptible genotype had higher constitutive expression. At 72 hpi, the susceptible genotype upregulated SA biosynthesis and signaling genes, such as numerous PR proteins, as well as genes in the JA pathway. Interestingly, isoflavone reductase (*IFR*) and phenylalanine ammonia lyase (*PAL*) were upregulated in the susceptible genotype, while the resistant genotype had higher constitutive expression in mock-inoculated samples (Additional file 1: Figure S5). These genes are known to participate in isoflavonoid biosynthesis of anti-fungal phytoalexins [55, 56].

**Table 4:** Differentially expressed genes in SA and JA pathways for the resistant and susceptible genotype

Hormone pathway	Comparison	Genes	Gene abbreviation	Function	log_2_FC
*SA biosynthesis and signaling*	Resistant inoculated vs. mock 72 hpi	MtrunA17_Chr7g0256321	*MtNOOT1*	NODULE ROOT 1	2.91
	Susceptible inoculated vs. mock 72 hpi	MtrunA17_Chr2g0295051	*MtPR10-3*	Pathogenesis-related protein 10	8.25
	Susceptible inoculated vs. mock 72 hpi	MtrunA17_Chr2g0295064	*MtPR10-6*	Pathogenesis-related protein 10	6.97
	Susceptible inoculated vs. mock 72 hpi	MtrunA17_Chr1g0190651	*MtPR4*	pathogenesis-related protein 4	6.21
	Susceptible inoculated vs. mock 72 hpi	MtrunA17_Chr1g0181091	*MtPAL*	Phenylalanine Ammonia Lyase	6.09
	Susceptible inoculated vs. mock 72 hpi	MtrunA17_Chr5g0404511	*MtIFR*	Isoflavone reductase	5.67
	Susceptible inoculated vs. mock 72 hpi	MtrunA17_Chr2g0295371	*AtPR-1-like*	pathogenesis-related protein 1	3.80
	Susceptible inoculated vs. mock 72 hpi	MtrunA17_Chr4g0067951	*MtPR10-5*	Pathogenesis-related protein 10	3.39
	Susceptible inoculated vs. mock 72 hpi	MtrunA17_Chr2g0295021	*MtPR10-2*	Pathogenesis-related protein 10	3.34
	Susceptible inoculated vs. mock 72 hpi	MtrunA17_Chr2g0295141	*MtPR10-4*	Pathogenesis-related protein 10	1.35
	Susceptible inoculated vs. mock 72 hpi	MtrunA17_Chr2g0295001	*MtPR10-1*	Pathogenesis-related protein 10-1	1.07
	Susceptible inoculated vs. mock 72 hpi	MtrunA17_Chr5g0397821	*MtPR5*	pathogenesis-related protein 5	1.03
	Susceptible inoculated vs. mock 72 hpi	MtrunA17_Chr1g0171771	*MtNOOT2*	NODULE ROOT 2	-1.21
*JA biosynthesis and signaling*	Resistant inoculated vs. mock 24 hpi	MtrunA17_Chr7g0272791	*MtLOX1-5*	Putative linoleate 9 S-lipoxygenase	1.72
	Resistant inoculated vs. mock 48 hpi	MtrunA17_Chr7g0272791	*MtLOX1-5*	Putative linoleate 9 S-lipoxygenase	1.19
	Resistant inoculated vs. mock 72 hpi	MtrunA17_Chr7g0272791	*MtLOX1-5*	Putative linoleate 9 S-lipoxygenase	1.09
	Susceptible inoculated vs. mock 72 hpi	MtrunA17_Chr3g0141271	*MtAOS2*	allene oxide synthase 2	4.37
	Susceptible inoculated vs. mock 72 hpi	MtrunA17_Chr5g0394861	*MtOPR*	12-OPDA reductase (TC94406)	2.99
	Susceptible inoculated vs. mock 72 hpi	MtrunA17_Chr1g0154381	*MtAOS1*	allene oxide synthase 1	2.42
	Susceptible inoculated vs. mock 72 hpi	MtrunA17_Chr8g0344201	*MtLOX6*	lipoxygenase (TC97760)	1.79
	Susceptible inoculated vs. mock 72 hpi	MtrunA17_Chr4g0070561	*MtOPR3*	oxophytodienoate-reductase 3	-1.25
	Susceptible inoculated vs. mock 72 hpi	MtrunA17_Chr4g0033171	*MtLOX1*	lipoxygenase (TC100514)	-3.62

### Contrasting expression profiles in QTL provide candidate genes for disease resistance

**Fig. 4:**
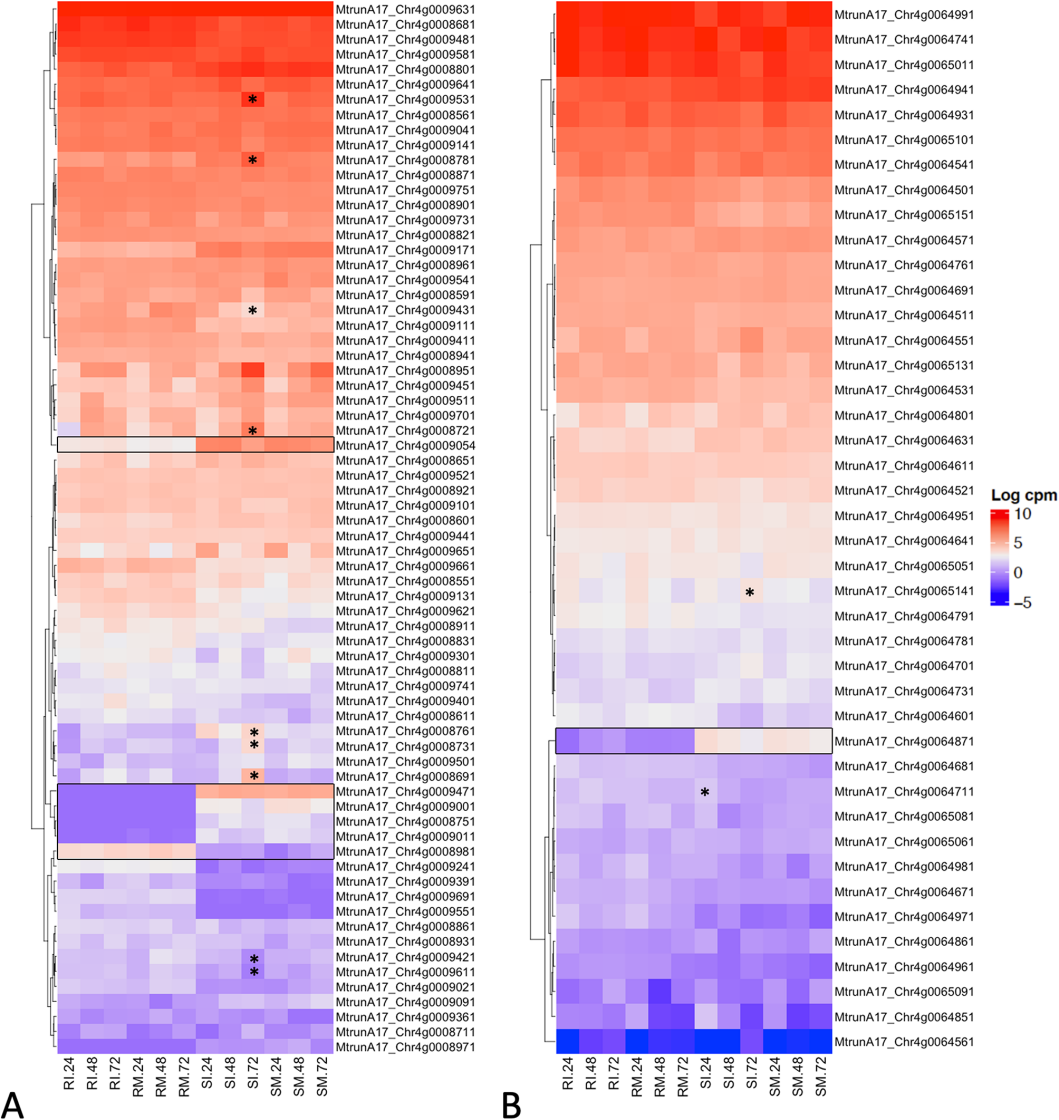
Gene expression profiles for QTL regions. Heatmaps are displayed in log_2_CPM for QTL (**A**) *rnpm1* and (**B**) *rnpm2*. Genes with contrasting expression profiles between resistant and susceptible genotypes are outlined with a box. Differentially expressed genes in specific tissues are indicated with asterisks. Sample ID abbreviations are SM: susceptible mock-inoculated, SI: susceptible inoculated, RM: resistant mock-inoculated, RI: resistant inoculated, followed by hours post inoculation (24, 48, or 72 hpi)

QTL regions *rnpm1* and *rnpm2* described by Kamphuis et al. [7] for SBS disease resistance were examined for differentially expressed genes. We examined the expression of 130 genes within a 1 Mbp region across *rnpm1*, and 69 genes across approximately 440 kbp in the fine-mapped *rnpm2* region [57]. Overall, differential expression of genes in these regions was only identified in the susceptible genotype (Additional file 2: Table S7). While no differential expression was observed across the QTL in the resistant genotype, there were genes with contrasting expression profiles between the resistant and susceptible genotypes (Fig. 4). In *rnpm1*, these include a Toll/Interleukin1 receptor-nucleotide binding site-leucine-rich repeat (TIR-NBS-LRR) disease resistance protein (MtrunA17_Chr4g0008981), which is constitutively expressed in HM078 at much higher levels than observed in A17. A Blast2GO annotation shows this gene has high similarity (78.79%) to the disease resistance protein RPS6 isoform X1. Conversely, TIR-NBS-LRRs (MtrunA17_Chr4g0009001, MtrunA17_Chr4g0009011) were expressed in A17 but had little to no expression in HM078. Finally, in *rnpm2*, a *PAM16*-like (MtrunA17_Chr4g0064871) gene was identified as having a contrasting expression profile between the resistant and susceptible genotype. Overall, genes with contrasting expression in the QTL regions for disease resistance may point to structural variation or transcriptional repression, and warrant further investigation.

### Plant immune system receptors feature notable candidate genes for disease resistance

We examined RLKs among the DEGs of both genotypes (Additional file 2: Table S8) (Additional file 1: Figure S6). For the resistant genotype, these included nine RLKs from five classes (Table 5). Of these, an RLK-Pelle-LRR was the most upregulated, and an RLK-Pelle-DLSV was upregulated over time. Neither were differentially expressed in the susceptible genotype. In the susceptible genotype, there were 166 differentially expressed RLK genes, and only three shared between both genotypes (Additional file 2: Table S8).

**Table 5:** Differentially expressed RLKs in the resistant genotype

Time point (hpi)	Gene ID	RLK class	log_2_FC	Unique to HM078
72	MtrunA17_Chr5g0436941	RLK-Pelle-LRR	4.62	Yes
48, 72	MtrunA17_Chr8g0361341	RLK-Pelle-DLSV	3.63, 3.23	Yes
72	MtrunA17_Chr1g0149711	RLK-Pelle-RLCK	3.00	Yes
72	MtrunA17_Chr4g0048711	RLK-Pelle-DLSV	2.50	No
72	MtrunA17_Chr1g0161701	RLK-Pelle-LRR	1.75	Yes
72	MtrunA17_Chr8g0358031	RLK-Pelle-DLSV	1.61	Yes
72	MtrunA17_Chr1g0160761	RLK-Pelle-LRK	1.56	Yes
72	MtrunA17_Chr1g0158251	RLK-Pelle-WAK	1.53	No
24	MtrunA17_Chr4g0071481	RLK-Pelle-RLCK	-2.22	No

Among the DEGs, we evaluated plant disease resistance genes (Additional file 1: Figure S7). In the resistant genotype, these included a TIR-NBS-LRR and three coiled-coil NLRs (Table 6). Notably, a CC-NBS plant disease resistance gene had the highest upregulation, with approximately a 75-fold increase in expression. This coiled-coil NLR gene has homology to *RPP13*-like protein 1 in *Pisum sativum* (amino acid identity = 73.35%), which lacks an LRR domain. A total of 64 plant disease resistance proteins were differentially expressed in the susceptible genotype, and none were shared between both genotypes (Additional file 2: Table S9).

**Table 6:** Differentially expressed NLR plant disease resistance genes in the resistant genotype

Time point (hpi)	Gene ID	Blast2GO Description	NLR class	log_2_FC	Unique to HM078
72	MtrunA17_Chr8g0392521	disease resistance RPP13-like protein 1	CC-NBS	6.25	Yes
72	MtrunA17_Chr6g0480181	TMV resistance protein N	TIR-NBS-LRR	3.57	Yes
48	MtrunA17_Chr6g0471941	disease resistance protein RGA2	CC-NBS-LRR	-1.32	Yes
72	MtrunA17_Chr3g0081391	disease resistance protein RPM1	CC-NBS-LRR	-4.24	Yes

### Selection of candidate genes for SBS disease resistance

After evaluating differential expression in a resistant and susceptible genotype of *M. truncatula* in response to *A. medicaginicola*, candidate genes for SBS disease resistance were selected (Table 7). Genes were chosen based on the following criteria: among the top ten upregulated or downregulated genes in the resistant genotype, upregulation across multiple time points in the resistant genotype, differentially expressed in the susceptible genotype with higher constitutive expression in the resistant genotype, shared DEGs between both genotypes with different expression levels, genes that had contrasting expression profiles in QTL regions, uniquely upregulated RLKs or NLRs in the resistant genotype, and upregulated genes in functionally enriched pathways. Finally, candidate genes that have not been linked to plant disease resistance in the literature were excluded. The expression profiles of candidate genes was visualized in a heatmap (Additional file 1: Figure S8).

**Table 7:** Candidate genes for SBS disease resistance in *M. truncatula* HM078

Gene ID (*Mt5.0*)	Gene	Rationale for selection	Potential role in defense	References
MtrunA17_Chr2g0327721	*MtKCS12*	Upregulated in resistant across multiple time points	VLCFAs contribute to physical barrier to pathogen	[39, 40]
MtrunA17_Chr8g0361341	RLK-Pelle-DLSV	Upregulated in resistant across multiple time points	Pathogen perception, activation of PR genes	[41, 42]
MtrunA17_Chr7g0272791	*MtLOX1-5*	Upregulated in resistant across multiple time points	JA biosynthesis, regulation of defense pathways	[37, 38]
MtrunA17_Chr5g0436941	RLK-Pelle-LRR	Highest upregulated RLK in resistant	Pathogen perception, activation of PR genes	[41, 42]
MtrunA17_Chr5g0404481	*MtIFR*	Upregulated in susceptible, constitutively expressed at higher levels in resistant	Isoflavonoid biosynthesis of anti-fungal phytoalexins	[55, 56]
MtrunA17_Chr1g0181091	*MtPAL*	Upregulated in susceptible, constitutively expressed at higher levels in resistant	Isoflavonoid biosynthesis of anti-fungal phytoalexins	[55, 56]
MtrunA17_Chr5g0443561	*MtSTR10-like*	Upregulated in resistant, downregulated in susceptible	Terpenoid biosynthesis of anti-fungal phytoalexins	[51, 52]
MtrunA17_Chr2g0323211	*MtPBP1*	Highly upregulated in enriched pathway in resistant	ROS signaling and defense pathway regulation	[31, 32]
MtrunA17_Chr4g0035371	MATH domain-containing protein	Highly upregulated in resistant	Regulates NLR turnover in *A. thaliana*	[35, 36]
MtrunA17_Chr2g0320061	*MtPrx28*	Highly upregulated and in enriched pathway in resistant	ROS response, DAMP elicited pathways	[33, 34]
MtrunA17_Chr8g0392521	*RPP13-like*	Highest upregulated NLR in resistant	NLR gene confers resistance	[16, 83]
MtrunA17_Chr4g0009001	TIR-NBS-LRR	Contrasting expression profile in QTL *rnpm1*	NLR acting as susceptibility factor	[7, 14]
MtrunA17_Chr4g0009011	TIR-NBS-LRR	Contrasting expression profile in QTL *rnpm1*	NLR acting as susceptibility factor	[7, 14]
MtrunA17_Chr4g0064871	*PAM16-like*	Contrasting expression profile in QTL *rnpm2*	PAM16 as negative regulator of plant immunity	[81, 82]
MtrunA17_Chr7g0263131	*MtZAT11*	Upregulated in both genotypes, higher upregulation in resistant	Regulator of hormone pathways in response to biotic stress	[53, 54]
MtrunA17_Chr5g0417371	*MtRNASET2*	Upregulated in enriched pathway in resistant	Inhibits pathogen colonization	[64, 65]
MtrunA17_Chr3g0122031	*MtXET*	Upregulated in enriched pathway in resistant	Cell wall strengthening	[66–68]
MtrunA17_Chr2g0330291	*MtBGAL8*	Upregulated in enriched pathway in resistant	Cell wall modification	[69, 70]
MtrunA17_Chr5g0402831	*MtAAO*	Upregulated in enriched pathway in resistant	ROS production and defense signaling	[71, 72]
MtrunA17_Chr0c27g0493921	*MtCTSH*	Highly downregulated in resistant	Regulates protein degradation	[43, 45]
MtrunA17_Chr4g0041901	*MtPPR*	Highly downregulated in resistant	Post-transcriptional regulation	[46, 48]
MtrunA17_Chr3g0081391	*MtRPM1-like*	Highly downregulated in resistant	Defense signaling	[49, 50]

### Validation of transcriptome sequencing data with qPCR

To validate our transcriptome sequencing results we performed qPCR on cDNA synthesized from the RNA samples collected throughout our experiment. We selected eight genes for validation across all time points for both genotypes. Relative quantification of target genes was performed compared to *MtACTIN11*, and RNA-seq fold change had a significant positive correlation (*R* = 0.8–0.98) with qPCR fold change for each gene tested (Additional file 1: Figure S9).

## Discussion

In this study, we analyzed the transcriptomes of both resistant and susceptible *M. truncatula* accessions in response to inoculation with the necrotrophic pathogen *A. medicaginicola* at three time points. We observed an approximate 24-hour delay in fungal penetration of the resistant genotype, possibly due to physical barriers or antimicrobial compounds [58, 59]. Invasive hyphae were more difficult to identify at earlier time points on both genotypes, likely resulting in less dramatic transcriptional shifts in whole-leaf samples and overlap between mock and inoculated samples observed on PCA plots at 24 and 48 hpi. Variability between individual plants and uneven inoculation application may have contributed to dispersal between biological replicates, particularly at 48 hpi, reducing statistical power and limiting our ability to detect differential expression. PCA analysis showed the largest degree of separation between mock and inoculated samples at 72 hpi, which corresponded to the highest numbers of DEGs for both genotypes. Similar studies have also noted large differences in DEGs between host genotypes in response to fungal pathogens, likely due to the number of invaded cells being sampled, highlighting a limitation of RNA-seq experiments [60].

### Transcriptomics of ascochyta blights reveal similar findings for several legume species

Ascochyta blights of multiple legume species have been previously investigated using similar RNA-seq studies [8, 61–63]. In *M. truncatula*, numerous PR proteins, as well as SA and JA hormone pathway genes were found to be upregulated at 12 hpi [8]. We identified upregulation of PR proteins, but primarily in the susceptible genotype. RNA-seq studies of ascochyta blight of chickpea, *Cicer arietinum*, have been conducted, and found that RLKs, NBS-NLRs, transcription factors, as well as SA, JA, ET, and ABA hormone pathway genes likely contribute to disease resistance [62, 63]. In our study, we found upregulation of genes in these families, including numerous transcription factor families, as well as unique NBS-NLRs and RLKs in the resistant genotype. Finally, Ascochyta blight of grass pea, *Lathyrus sativus*, was investigated using transcriptomics, which described candidate genes for disease resistance in the SA, ET, and ABA hormone pathways, as well as cell wall remodeling genes, peroxidase, PR proteins, and detoxifying genes [61]. Our findings align with previous studies in relation to the functional description of many candidate genes for disease resistance. Both cell wall remodeling and ROS response genes were found to be important factors for resistance to *A. medicaginicola*. However, we also identified candidate genes that were not highlighted in previous studies, such as calcium-binding protein *MtPBP1* and VLCFA synthesis gene *MtKCS12*.

### **Functional enrichment in the resistant genotype suggest antifungal mechanisms**

Functional enrichment analysis of DEGs revealed differences in the host response of resistant and susceptible genotypes to *A. medicaginicola*. In the resistant genotype, significantly enriched terms related to the extracellular region, cell wall, and calcium ion binding, which were all unique to the resistant genotype. Upregulated DEGs in extracellular region included peroxidase 28 (*Prx28*) and ribonuclease T2 (*RNASET2*). *Prx28* regulates redox signaling pathways for defense responses including cell wall thickening and PCD [33, 34]. *RNASET2* is thought to inhibit pathogen colonization at infection sites [64, 65]. Upregulated DEGs in the cell wall included xyloglucan/xyloglucosyl transferase (*XET*), β-galactosidase (*BGAL*), and ascorbate oxidase (*AAO*). *XET* cross-links xyloglucans to strengthen the cell wall [66–68]. *BGAL* hydrolyze β-galactosides to modify the cell wall [69, 70]. *AAO* produces ROS that mediate defense signaling [71, 72]. In the resistant genotype, the most upregulated DEG overall was PINOID-BINDING PROTEIN 1 (PBP1), which similar to calmodulin (*CML*), contains domains that bind calcium ions [73, 74]. Calcium ion elevations activate MAPK signaling cascades, the oxidative burst, and the hypersensitive response [75–77]. Resistant *M. truncatula* genotype HM078 has been observed to have a hypersensitive-like response after inoculation with *A. medicaginicola* that could be attributed to an oxidative burst [7]. Overall, the function of genes in these pathways shed light on potential antifungal mechanisms in the resistant genotype.

#### Plant hormone pathways engaged during the host response to *A. medicaginicola*

Plant hormones, such as SA and JA, are crucial signaling molecules to regulate defense responses [78]. JA is synthesized from fatty acids in the octadecanoid pathway by enzymes including OPDA reductase, lipoxygenase, allene oxide synthase, and lipase [79]. JA signaling mediates defense responses against necrotrophic fungi, resulting in lignin formation, synthesis of PR proteins, flavonoids, terpenoids, and phytoalexins [80]. In the resistant genotype, *MtLOX1-5*, was upregulated from 24 to 72 hpi, while the susceptible genotype, upregulated JA biosynthesis genes at 72 hpi. On the other hand, SA is synthesized from phenylalanine in the phenylpropanoid pathway by a series of enzymes resulting in isoflavonoid phytoalexins, lignin, benzoic acid, phenylpropenes, and coumarins [56]. Chalcone synthase (*CHS*), isoflavone reductase (*IFR*), 4-coumarate-CoA ligase (*4CL*), and phenylalanine ammonia lyase (*PAL*) mediate flavonoid biosynthesis. In the susceptible genotype, phenylpropanoid pathway genes were enriched, including *MtCHS-1 A*, *MtCHS-1B*, *MtIFR*, *MtPAL*, and *Mt4CL-2*. Kamphuis et al. [8] found an induction of SA in resistant and susceptible genotypes, but found the resistant genotype HM078 contained constitutively higher levels of isoflavonoids. This was supported by our finding that *MtPAL* and *MtIFR* were upregulated at 72 hpi in the susceptible genotype, however, the resistant genotype had higher constitutive expression. Overall, both SA and JA likely play crucial roles as signaling molecules and the host response to *A. medicaginicola*.

#### Candidate genes in QTL identified based on contrasting expression profiles

QTL *rnpm1* and *rnpm2* were examined for their role in SBS disease resistance, focusing on gene expression patterns. DEGs were only detected at 72 hpi in the susceptible genotype. These QTL are known to be inherited recessively, which may support the inverse gene-for-gene model [7]. This paradigm is illustrated by the *LOV1* gene in oat that confers sensitivity to the fungal toxin victorin, while also providing resistance to the crown rust fungus [15]. Genes expressed in the susceptible genotype and not expressed in the resistant genotype are of particular interest. In *rnpm1*, TIR-NBS-NLR genes MtrunA17_Chr4g0009001 and MtrunA17_Chr4g0009011 were found to be expressed only in the susceptible genotype, supporting this concept. In *rnpm2*, which does not contain NLRs, a gene orthologous to *AtPAM16* showed no expression in the resistant genotype and high expression in the susceptible genotype. *PAM16* is known to play a role in plant immunity, as shown in *A. thaliana* mutants lacking this gene, which exhibit enhanced disease resistance [81, 82]. Backcrossing studies with an *AtPam16* knockout mutant (*muse5-1*) indicated that the recessive inheritance of the resistant phenotype aligns with the recessive inheritance pattern observed for *rnpm2*. Overall, genes with differing expression patterns between the resistant and susceptible genotypes in these QTL regions are candidate genes for further study.

***RPP13*-like plant disease protein likely involved in incompatible host response**.

A promising plant disease resistance gene, potentially involved in the incompatible host response was identified. This coiled-coil class NLR gene was uniquely upregulated in the resistant genotype. Notably, this disease resistant protein is similar to *RPP13*-like protein 1, which confers broad spectrum resistance to biotrophic pathogens *Melampsora lini* (flax rust), as well as *Hyaloperonospora arabidopsidis* and *Peronospora parasitica* (downy mildew) in *A. thaliana* [83, 84]. Coiled-coil class NLRs have also been shown to play important roles in plant immunity to necrotrophic pathogens, for example, overexpression of *GbCNL130* confers resistance to *Verticillium dahliae* in cotton [85]. Pathogen ligand binding to these proteins results in downstream defense responses. For instance, the activation of HOPZ-ACTIVATED RESISTANCE 1 (ZAR1) causes a calcium ion influx, ROS production, and cell death conferring resistance to *Pseudomonas syringae* in *A. thaliana* [86, 87]. Future directions will include investigating the specific role of this coiled-coil class NLR.

## Conclusion

Examining host-pathogen interactions between *M. truncatula* and the necrotrophic fungal pathogen *A. medicaginicola* has the potential to illuminate molecular factors that could be used to enhance disease resistance to Ascochyta blights in legumes. We performed a transcriptome analysis for a resistant (HM078) and susceptible (A17) *M. truncatula* genotype infected with *A. medicaginicola* to evaluate the host response and identify candidate genes for disease resistance. We examined DEGs, functionally enriched pathways, hormone pathways, RLKs, NLRs, and QTL regions for SBS disease resistance. We identified a number of candidate genes for disease resistance with support from the literature. After functional validation of candidate genes, future studies will explore engineering SBS disease resistance in the economically important forage crop alfalfa.

### Electronic supplementary material

Below is the link to the electronic supplementary material.

Supplementary Material 1

Supplementary Material 2

## Data Availability

All raw sequence data has been deposited in the NCBI database under BioProject PRJNA975868. SRA numbers SRR24775309, SRR24775310, SRR24775311, SRR24775312, SRR24775313, SRR24775314, SRR24775315, SRR24775316, SRR24775317, SRR24775318, SRR24775319, SRR24775320, SRR24775321, SRR24775322, SRR24775326, SRR24775327, SRR24775328, SRR24775329, SRR24775330, SRR24775331, SRR24775332, SRR24775333, SRR24775334, SRR24775338, SRR24775339, SRR24775341, SRR24775342, SRR24775343, SRR24775344, SRR24775345, SRR24775349, SRR24775350, SRR24793325, SRR24793326, SRR24793327, SRR24793328 contain the RNA-seq reads used throughout this study. The code run throughout this study (RNA-seq_associated_code.html) and the RNA-seq feature count data (feature_count_matrix.txt) is available on GitHub (https://github.com/shaun-curtin/RNA-seq-analysis-of-Spring-Black-Stem-Disease-SBS-). Germplasm of *M. truncatula* used in this study can be requested at (https://medicago.legumeinfo.org/tools/germplasm/).

## Abbreviations

SBS: Spring black stem
SARDI: South Australian Research and Development Institute
QTL: Quantitative trait locus
hpi: Hours post inoculation
PR: Pathogenesis-related
rnpm1: Resistance to the necrotroph Phoma medicaginis one
rnpm2: Resistance to the necrotroph Phoma medicaginis two
PCD: Programmed cell death
PAMPs: Pathogen associated molecular patterns
PRR: Pattern recognition receptors
RLK: Receptor-like kinases
RLP: Receptor-like proteins
PTI: PAMP-triggered immunity
ROS: Reactive oxygen species
NLR: Nucleotide-binding site and leucine-rich repeat
ETI: Effector-triggered immunity
ETS: Effector-triggered susceptibility
SA: Salicylic acid
JA: Jasmonic acid
ABA: Abscisic acid
ET: Ethylene
DEGs: Differentially expressed genes
qPCR: Quantitative RT-PCR
DI: De-ionized
PDA: Potato dextrose agar
GO: Gene Ontology
BP: Biological Processes
MF: Molecular Function
CC: Cellular Component
KEGG: Kyoto Encyclopedia of Genes and Genomes
PCA: Principal component analysis
CPM: Counts per million
VLCFA: Very long chain fatty acids
CHS: Chalcone synthase
MFS: Major facilitator superfamily
IFR: Isoflavone reductase
PAL: Phenylalanine ammonia lyase
TIR-NBS-LRR: Toll/Interleukin1 receptor-nucleotide binding site-leucine-rich repeat
Prx28: Peroxidase 28
RNASET2: Ribonuclease T2
XET: Xyloglucan/xyloglucosyl transferase
BGAL: β-galactosidase
AAO: Ascorbate oxidase
PBP1: Pinoid-Binding Protein 1
CML: Calmodulin
4CL: 4-coumarate-CoA ligase
LOV1: Locus Orchestrating Victorin Effects1
PAM16: Presequence translocase-associated motor 16
ZAR1: Hopz-Activated Resistance 1
