# Correction: Transcriptome analysis of resistant and susceptible Medicago truncatula genotypes in response to spring black stem and leaf spot disease

**DOI:** 10.1186/s12870-024-05493-8

**Published:** 2024-08-14

**Authors:** Jacob R. Botkin, Shaun J. Curtin

**Affiliations:** 1 Plant Science Research Unit, United States Department of Agriculture, St Paul, MN 55108 USA; 2 Department of Plant Pathology, University of Minnesota, St. Paul, MN 55108 USA; 3 Department of Agronomy and Plant Genetics, University of Minnesota, St. Paul, MN 55108 USA; 4 Center for Plant Precision Genomics, University of Minnesota, St. Paul, MN 55108 USA; 5 Center for Genome Engineering, University of Minnesota, St. Paul, MN 55108 USA

**Correction:** **BMC Plant Biol 24, 720 (2024)**

**https://doi.org/10.1186/s12870-024-05444-3**

Following publication of the original article [1], the authors identified errors in the labelling of figures. During the proofing process, author sent an email concerning the incorrect label of figures. Unfortunately, the email was not received.

The correct figures are presented below:

**Incorrect Figure** 1 **:**

**Fig. 1:**
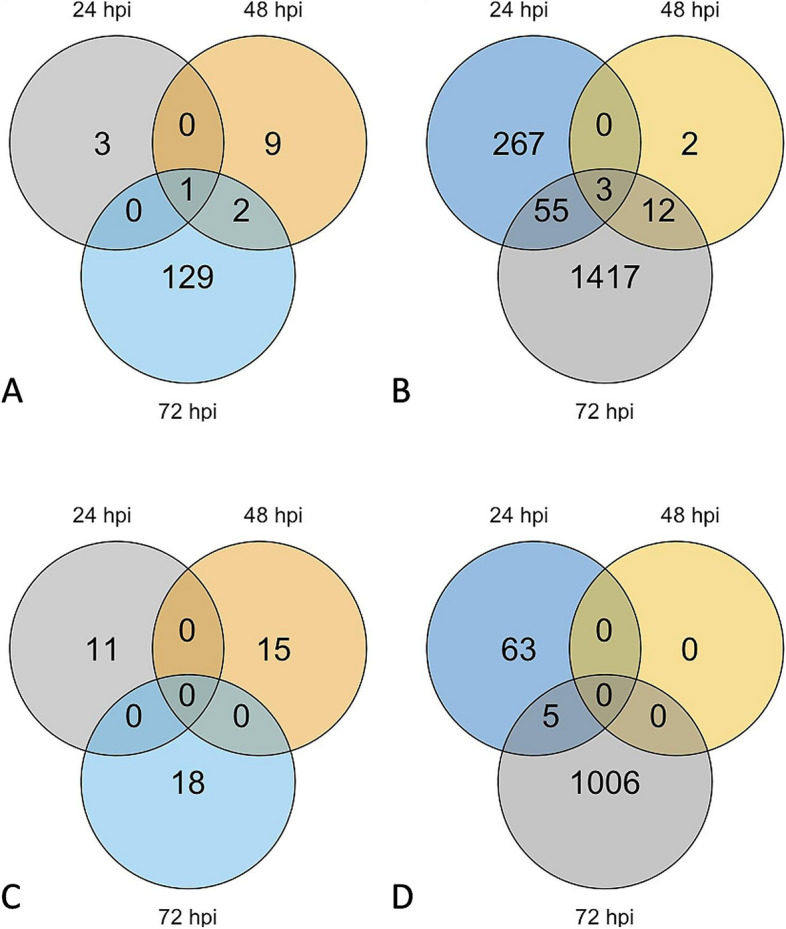
Cross sections of *M. truncatula* leaves infected with *A. medicaginicola*. Images were taken under GFP fluorescence (left) and RGB (right) for susceptible genotype A17 at (**A**) 24 hpi, (**B**) 48 hpi, and (**C**) 72 hpi, as well as the resistant genotype HM078 at (**D**) 24 hpi, (**E**) 48 hpi, and (**F**) 72 hpi. Red arrows indicate invasive hyphae penetrating leaf epidermal cells. Scale bars for (**A-F**) are 75, 50, 150, 50, 150, and 150 µm, respectively

**Correct Figure** 1

**Fig. 1:**
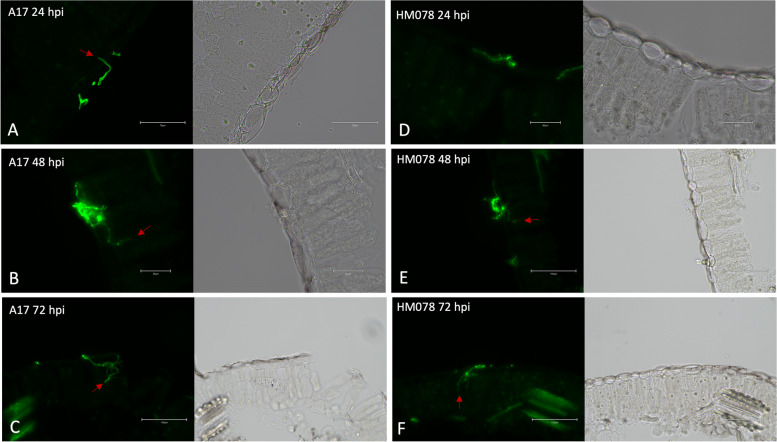
Cross sections of *M. truncatula* leaves infected with *A. medicaginicola*. Images were taken under GFP fluorescence (left) and RGB (right) for susceptible genotype A17 at (**A**) 24 hpi, (**B**) 48 hpi, and (**C**) 72 hpi, as well as the resistant genotype HM078 at (**D**) 24 hpi, (**E**) 48 hpi, and (**F**) 72 hpi. Red arrows indicate invasive hyphae penetrating leaf epidermal cells. Scale bars for (**A-F**) are 75, 50, 150, 50, 150, and 150 µm, respectively

**Incorrect Figure** 2

**Fig. 2:**
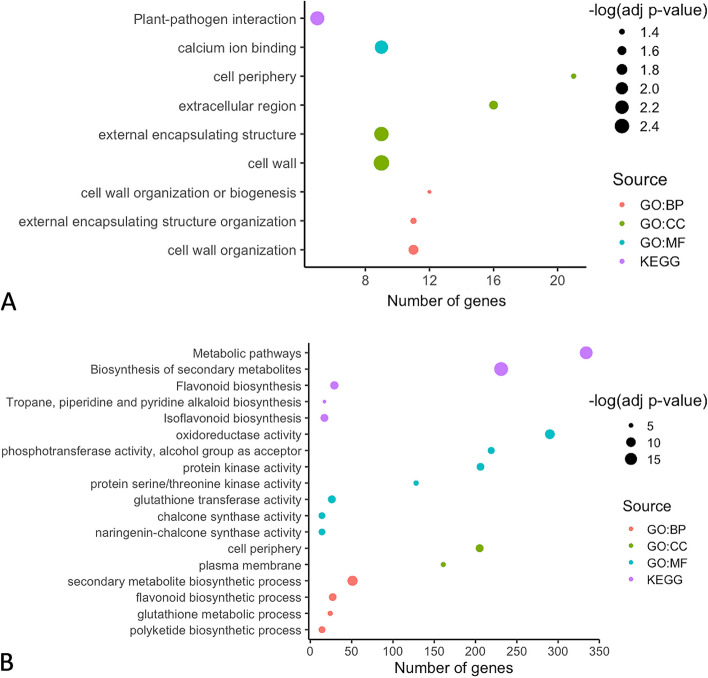
Number of DEGs for resistant and susceptible *M. truncatula* in response to *A. medicaginicola*. Venn diagrams of (**A**) Upregulated DEGs of resistant genotype HM078, (**B**) Upregulated DEGs of susceptible genotype A17, (**C**) Downregulated DEGs of resistant genotype HM078, and (**D**) Downregulated DEGs of susceptible genotype A17

**Correct Figure **2

**Fig. 2:**
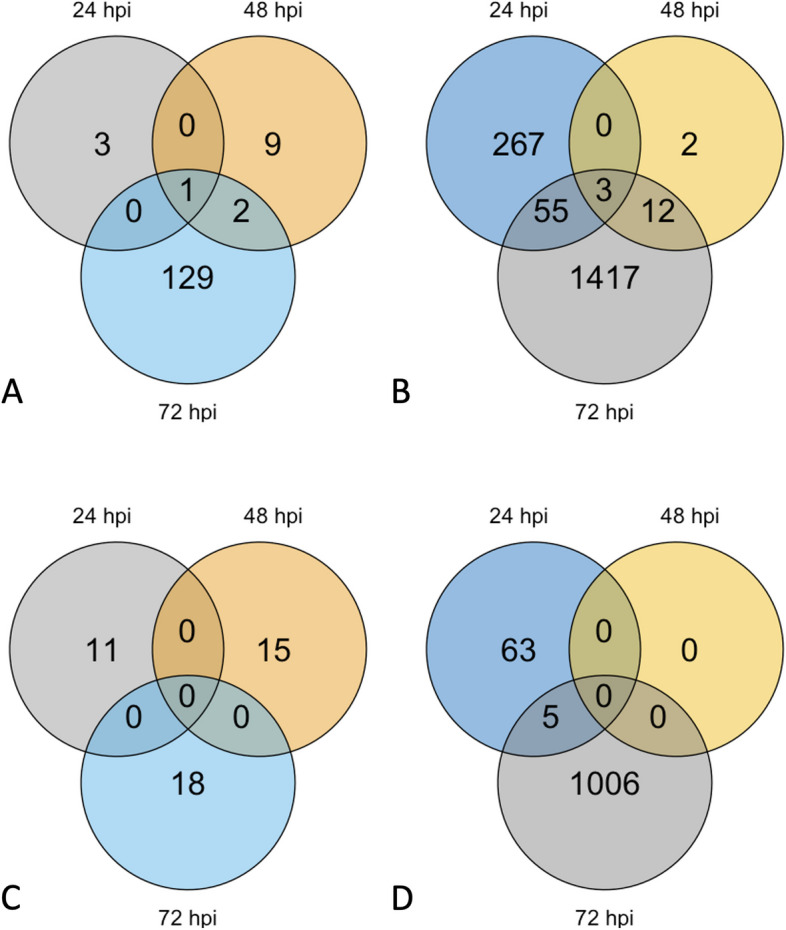
Number of DEGs for resistant and susceptible *M. truncatula* in response to *A. medicaginicola*. Venn diagrams of (**A**) Upregulated DEGs of resistant genotype HM078, (**B**) Upregulated DEGs of susceptible genotype A17, (**C**) Downregulated DEGs of resistant genotype HM078, and (**D**) Downregulated DEGs of susceptible genotype A17

**Incorrect** **Figure** 3

**Fig. 3:**
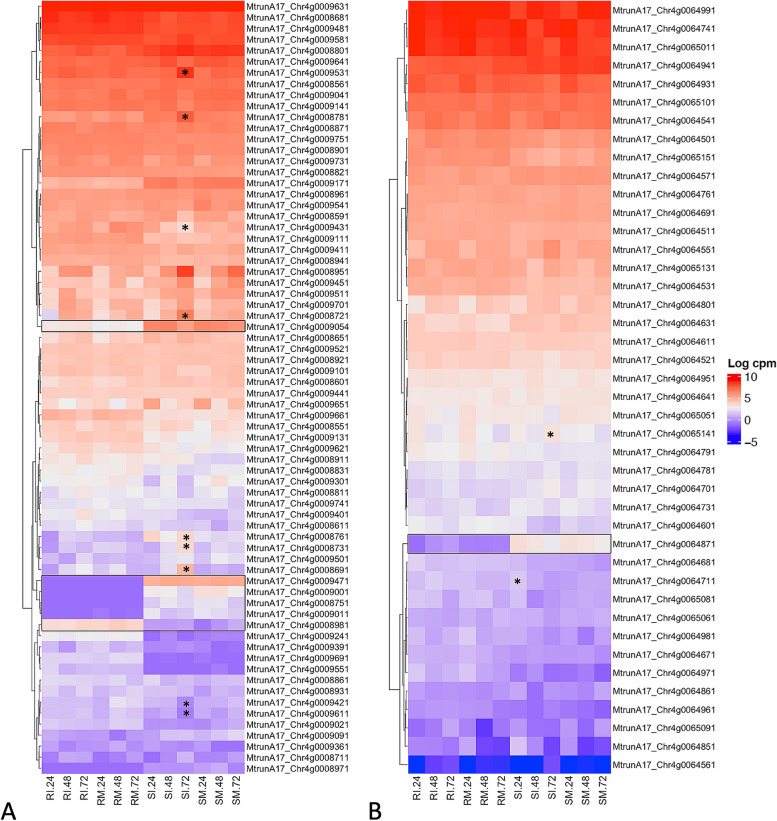
Functional enrichment analysis of resistant and susceptible *M. truncatula* in response to *A. medicaginicola*. Significantly enriched GO terms were analyzed for (**A**) DEGs in the resistant genotype HM078, and (**B**) DEGs in the susceptible genotype A17. Upregulated and downregulated DEGs across all time points were included for each genotype. GO (Gene Ontology) terms were grouped by Biological Processes (BP), Molecular Function (MF), Cellular Component (CC), or Kyoto Encyclopedia of Genes and Genomes (KEGG) pathways

**Correct** **Figure **3

**Fig. 3:**
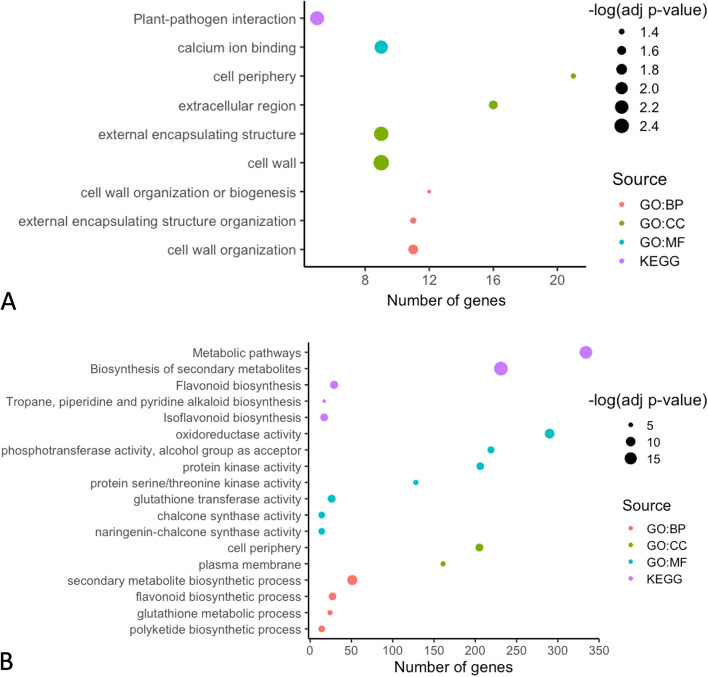
Functional enrichment analysis of resistant and susceptible *M. truncatula* in response to *A. medicaginicola*. Significantly enriched GO terms were analyzed for (**A**) DEGs in the resistant genotype HM078, and (**B**) DEGs in the susceptible genotype A17. Upregulated and downregulated DEGs across all time points were included for each genotype. GO (Gene Ontology) terms were grouped by Biological Processes (BP), Molecular Function (MF), Cellular Component (CC), or Kyoto Encyclopedia of Genes and Genomes (KEGG) pathways

**Incorrect** **Figure **4

**Fig. 4:**
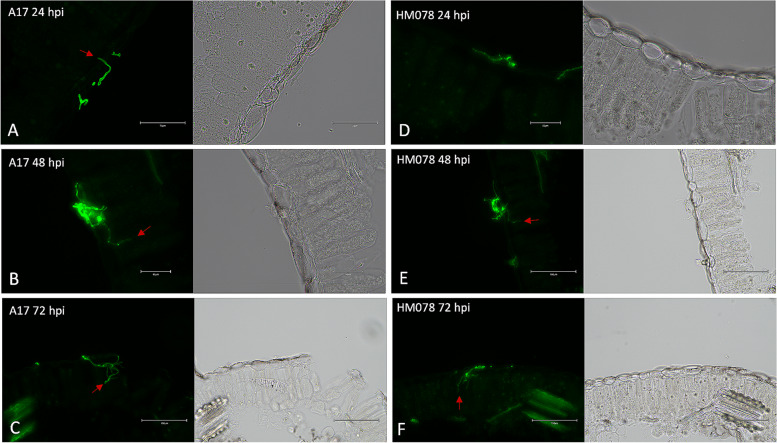
Gene expression profiles for QTL regions. Heatmaps are displayed in log_2_CPM for QTL (**A**) *rnpm1* and (**B**) *rnpm2*. Genes with contrasting expression profiles between resistant and susceptible genotypes are outlined with a box. Differentially expressed genes in specific tissues are indicated with asterisks. Sample ID abbreviations are SM: susceptible mock-inoculated, SI: susceptible inoculated, RM: resistant mock-inoculated, RI: resistant inoculated, followed by hours post inoculation (24, 48, or 72 hpi)

**Correct** **Figure **4

**Fig. 4:**
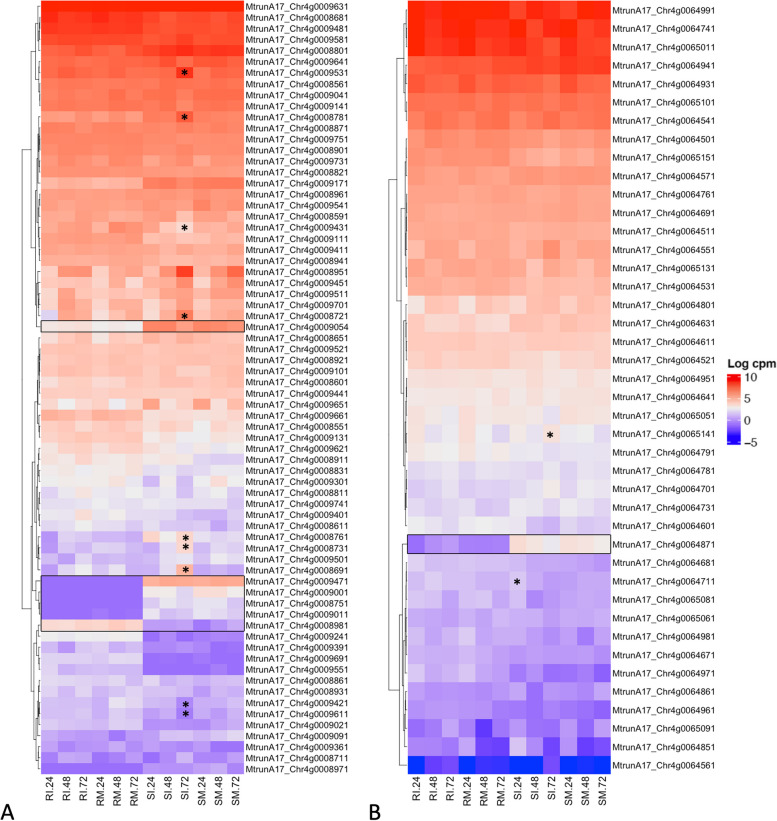
Gene expression profiles for QTL regions. Heatmaps are displayed in log_2_CPM for QTL (**A**) *rnpm1* and (**B**) *rnpm2*. Genes with contrasting expression profiles between resistant and susceptible genotypes are outlined with a box. Differentially expressed genes in specific tissues are indicated with asterisks. Sample ID abbreviations are SM: susceptible mock-inoculated, SI: susceptible inoculated, RM: resistant mock-inoculated, RI: resistant inoculated, followed by hours post inoculation (24, 48, or 72 hpi)

The original article [1] has been corrected.
