# Supplementary material for: Transcriptome analysis of resistant and susceptible Medicago truncatula genotypes in response to spring black stem and leaf spot disease
Source: BMC Plant Biol. 2024 Jul 29;24:720. doi: 10.1186/s12870-024-05444-3 (PMC11285230; doi:10.1186/s12870-024-05444-3)
Supplement: Supplementary file 2 — Supplementary Material 2 [file 12870_2024_5444_MOESM2_ESM.docx]

**
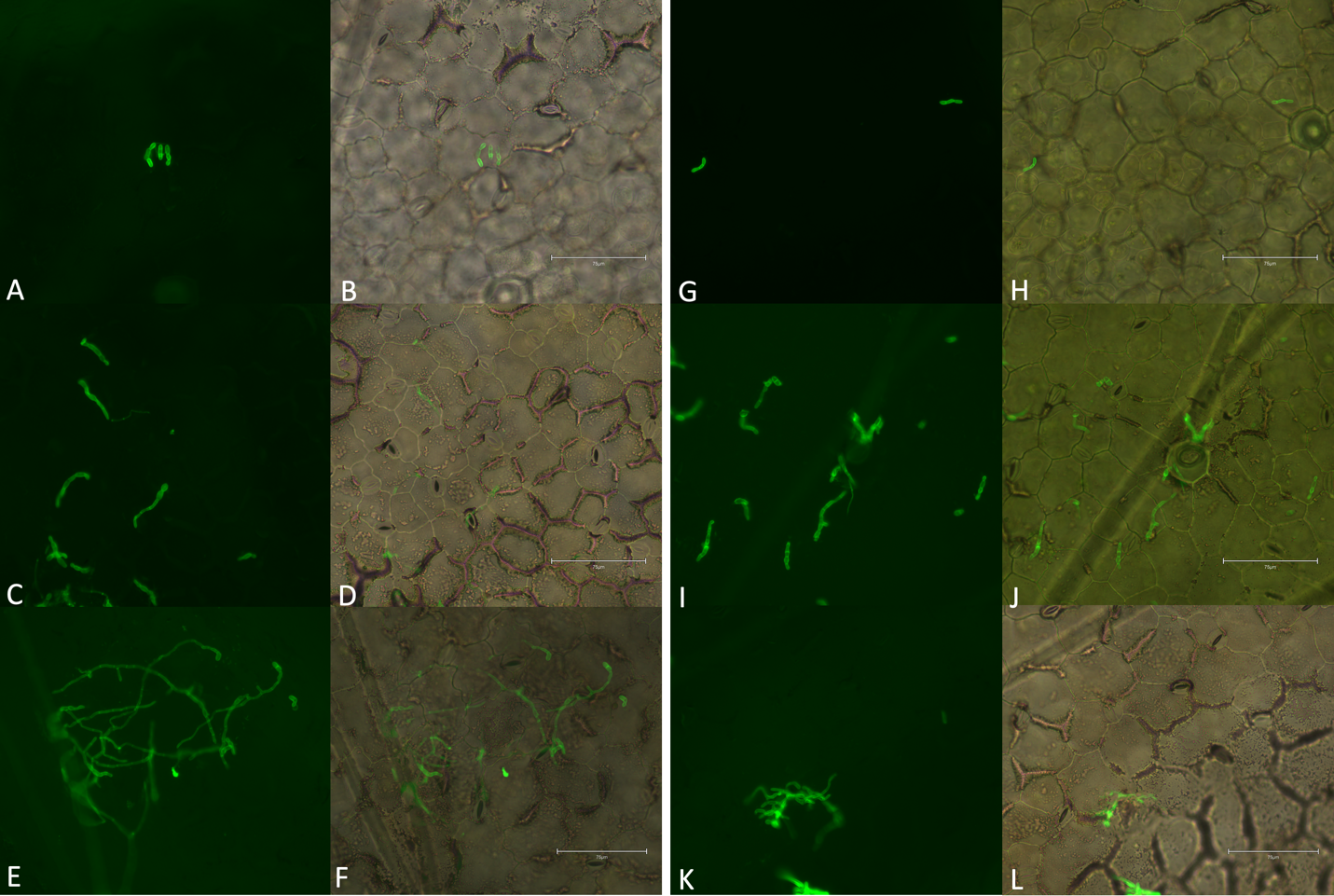
**

**Figure S1.** *A. medicaginicola* hyphal growth on inoculated leaf surface of *M. truncatula*. Images were taken under GFP fluorescence and overlaid on an RGB image of the susceptible genotype A17 at (A-B) 24 hpi, (C-D) 48 hpi, and (E-F) 72 hpi, as well as the resistant genotype HM078 at (G-H) 24 hpi, (I-J) 48 hpi, and (K-L) 72 hpi.


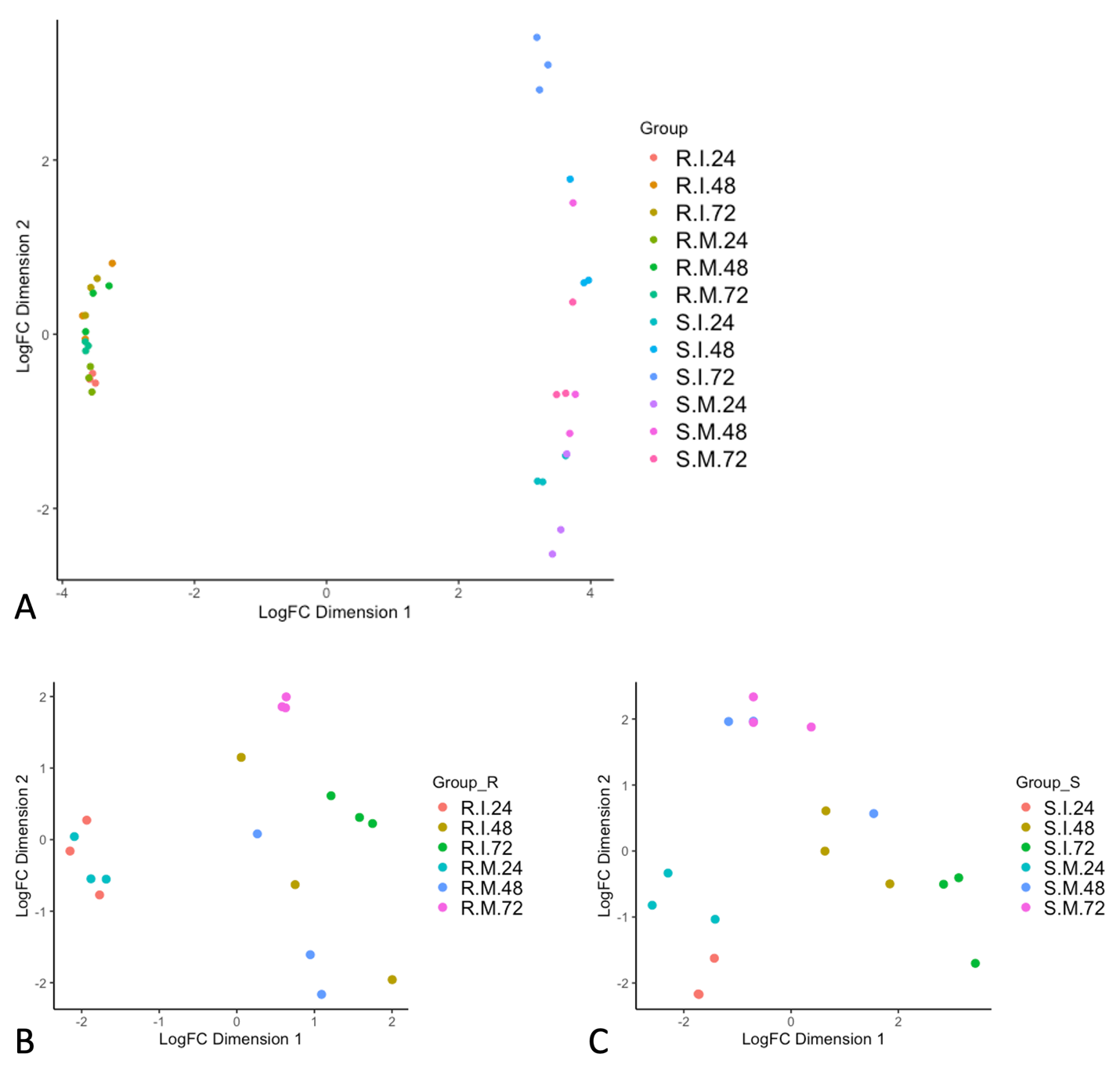


**Figure S2.** PCA plots of the biological coefficient of variation. PCA plots of RNA-seq samples for (A) resistant (HM078) and susceptible (A17) genotypes together, (B) the resistant genotype, and (C) the susceptible genotype. Sample naming conventions are R: resistant, S: susceptible, I: inoculated, M: mock-inoculated, followed by 24, 48, or 72 to indicate the time point.


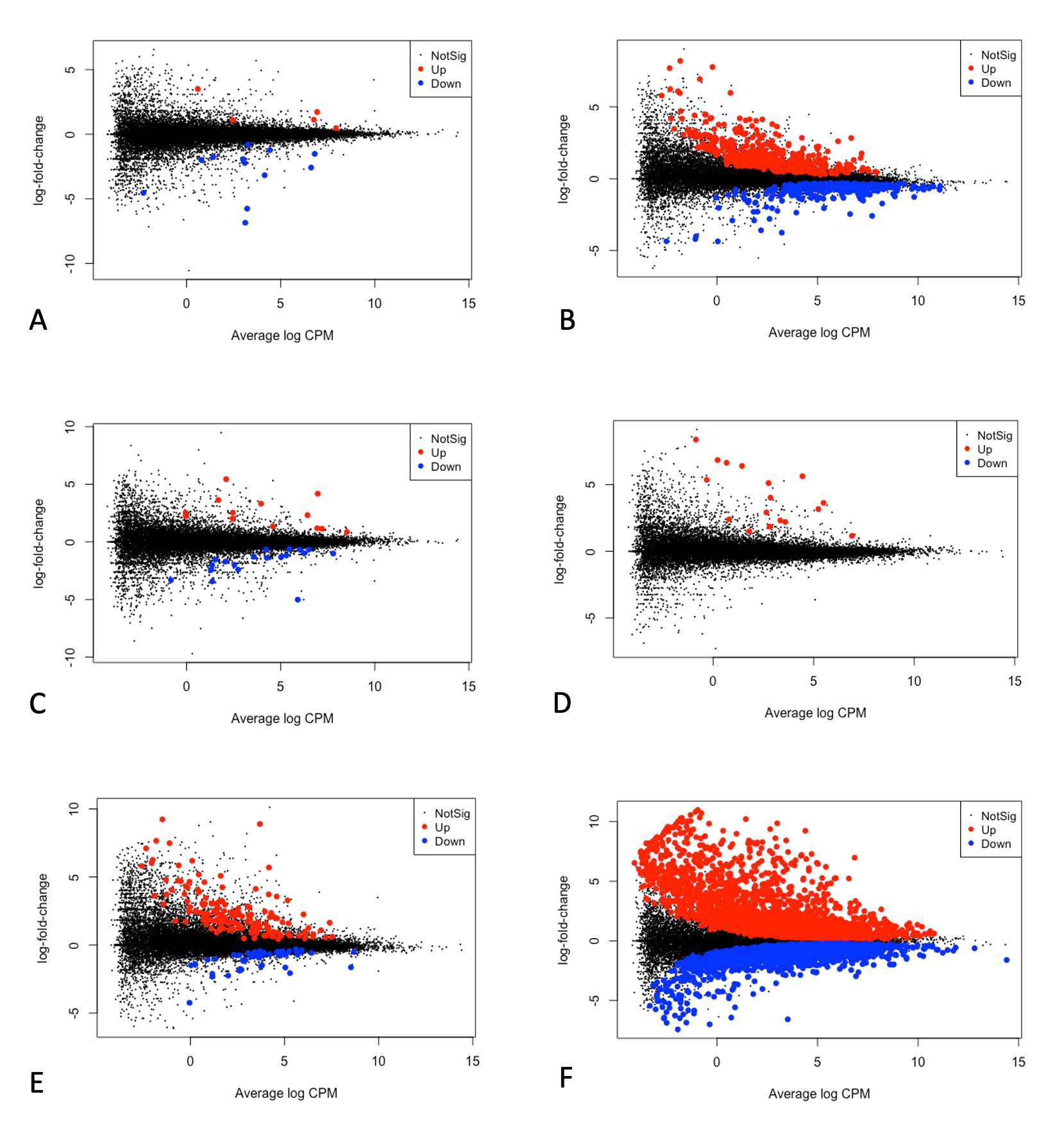


**Figure S3.** DEGs of HM078 and A17 at 24, 48, and 72 hours post inoculation (hpi). Statistical comparisons were inoculated versus mock inoculated within each accession at each time point. Volcano diagrams of (A) DEGs of HM078 at 24 hpi, (B) DEGs of A17 at 24 hpi, (C) DEGs of HM078 at 48 hpi, (D) DEGs of A17 at 48 hpi, (E) DEGs of HM078 at 72 hpi, and (F) DEGs of A17 at 72 hpi.


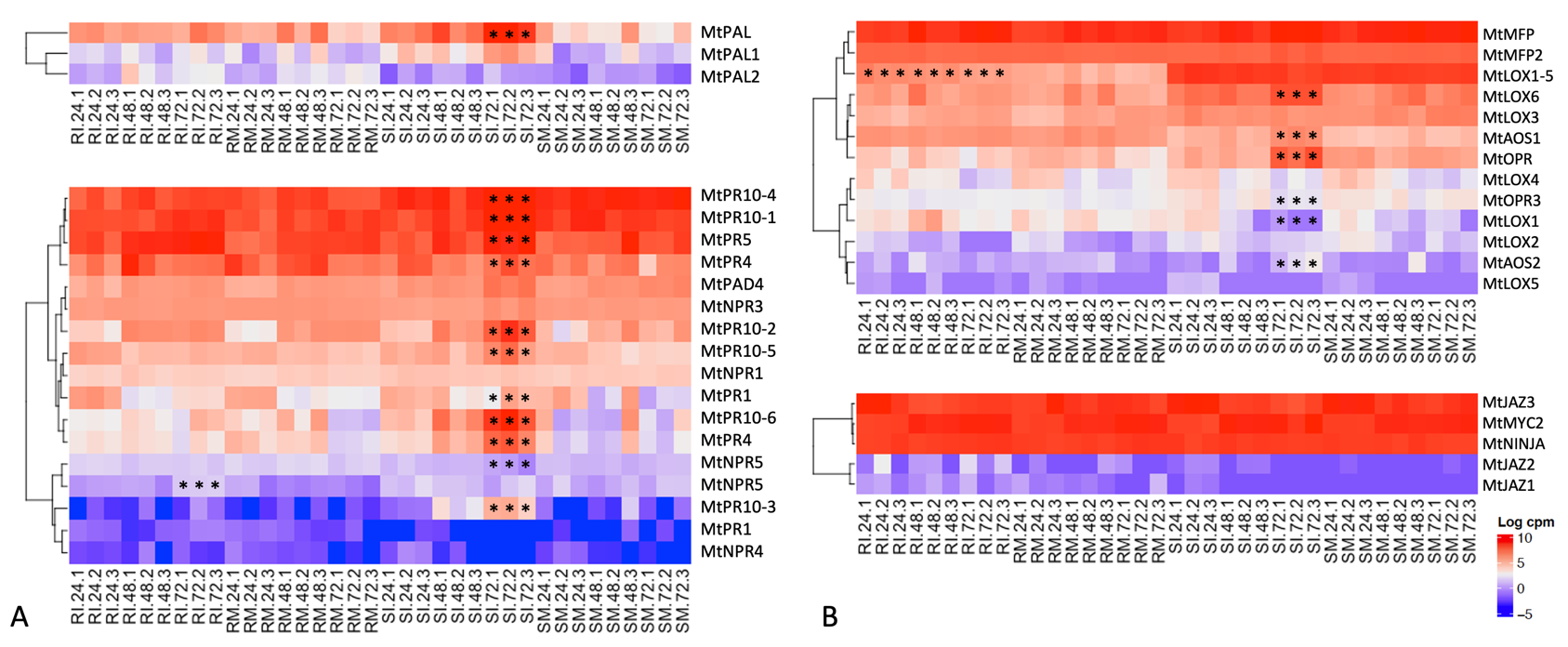


**Figure S4.** Gene expression profiles for salicylic acid (SA) and jasmonic acid (JA) pathway genes. Heatmaps are displayed in log_2_CPM for genes involved in (A) SA biosynthesis (top) and SA signaling (bottom), and (B) JA biosynthesis (top) and JA signaling (bottom). Differentially expressed genes indicated with asterisks. Sample ID abbreviations are SM: susceptible mock-inoculated, SI: susceptible inoculated, RM: resistant mock-inoculated, RI: resistant inoculated, followed by hours post inoculation (24, 48, or 72 hpi) and biological replicate (1, 2, or 3).


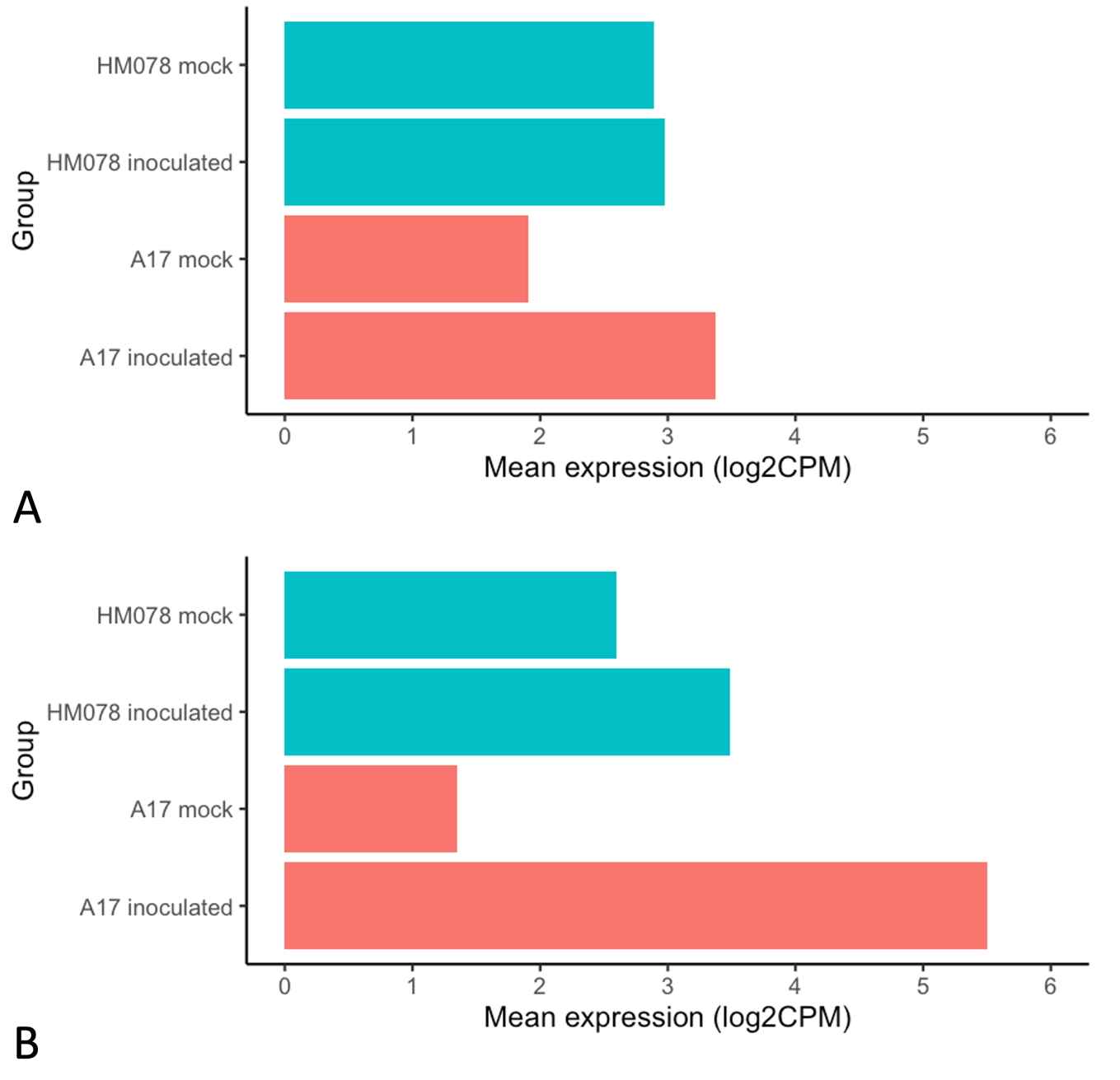


**Figure S5.** Mean expression of SA biosynthesis and signaling genes. (A) Isoflavone reductase (MtrunA17_Chr5g0404511) and (B) phenylalanine ammonia lyase (MtrunA17_Chr1g0181091) are upregulated in the susceptible genotype, but have higher constitutive expression in the resistant genotype.

**
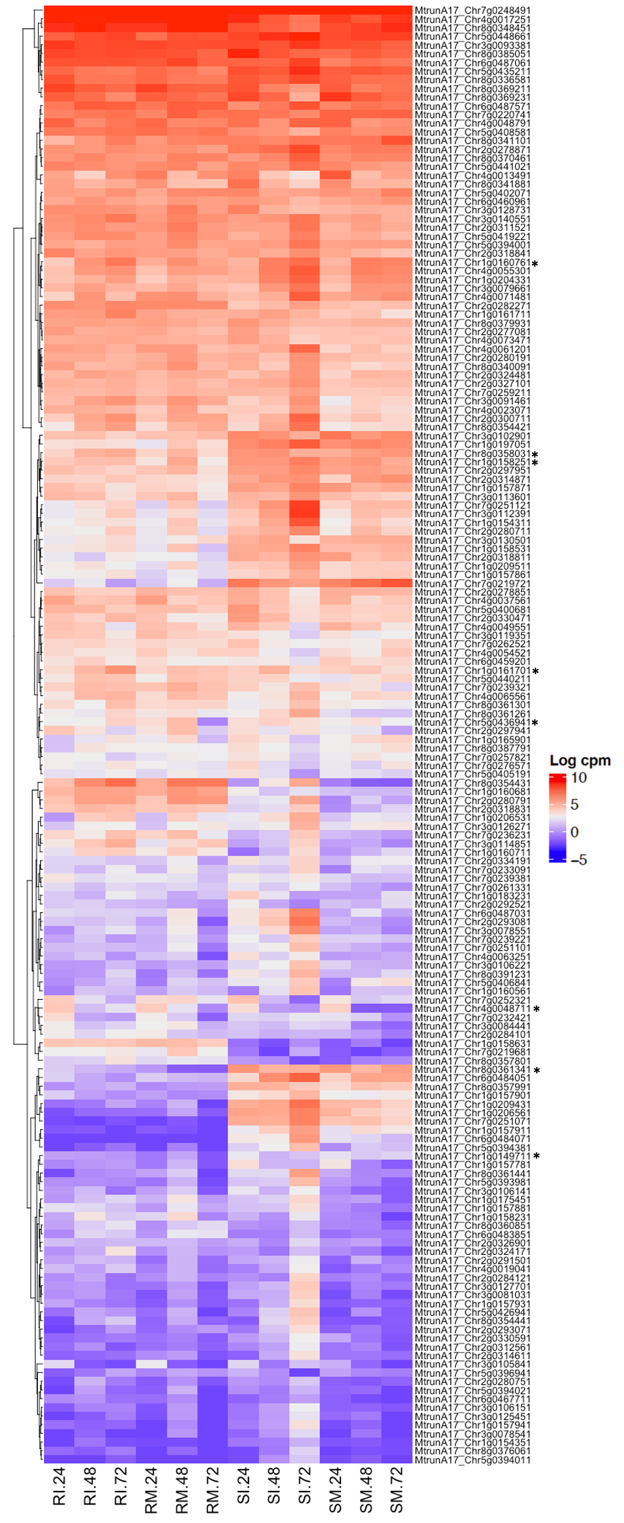
**

**Figure S6.** RLK expression profiles among DEGs for HM078 and A17. Differentially expressed genes in resistant genotype HM078 are marked with an asterisk. Sample ID abbreviations are SM: susceptible mock-inoculated, SI: susceptible inoculated, RM: resistant mock-inoculated, RI: resistant inoculated, followed by hours post inoculation (24, 48, or 72 hpi) and biological replicate (1, 2, or 3).


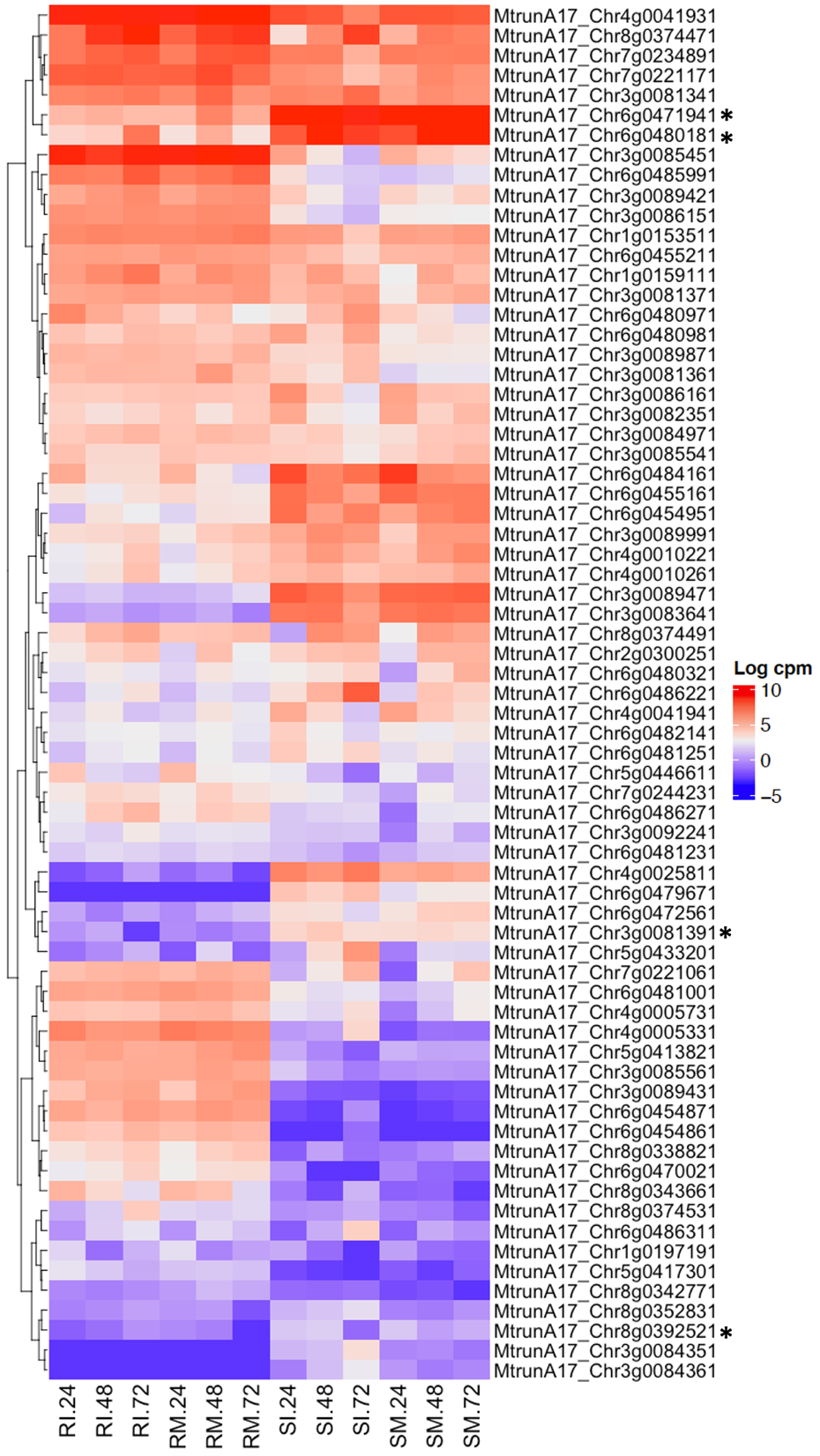


**Figure S7.** Plant disease resistance gene expression profiles for DEGs of HM078 and A17. DEGs in the resistant genotype HM078 are marked with an asterisk. Sample ID abbreviations are SM: susceptible mock-inoculated, SI: susceptible inoculated, RM: resistant mock-inoculated, RI: resistant inoculated, followed by hours post inoculation (24, 48, or 72 hpi).


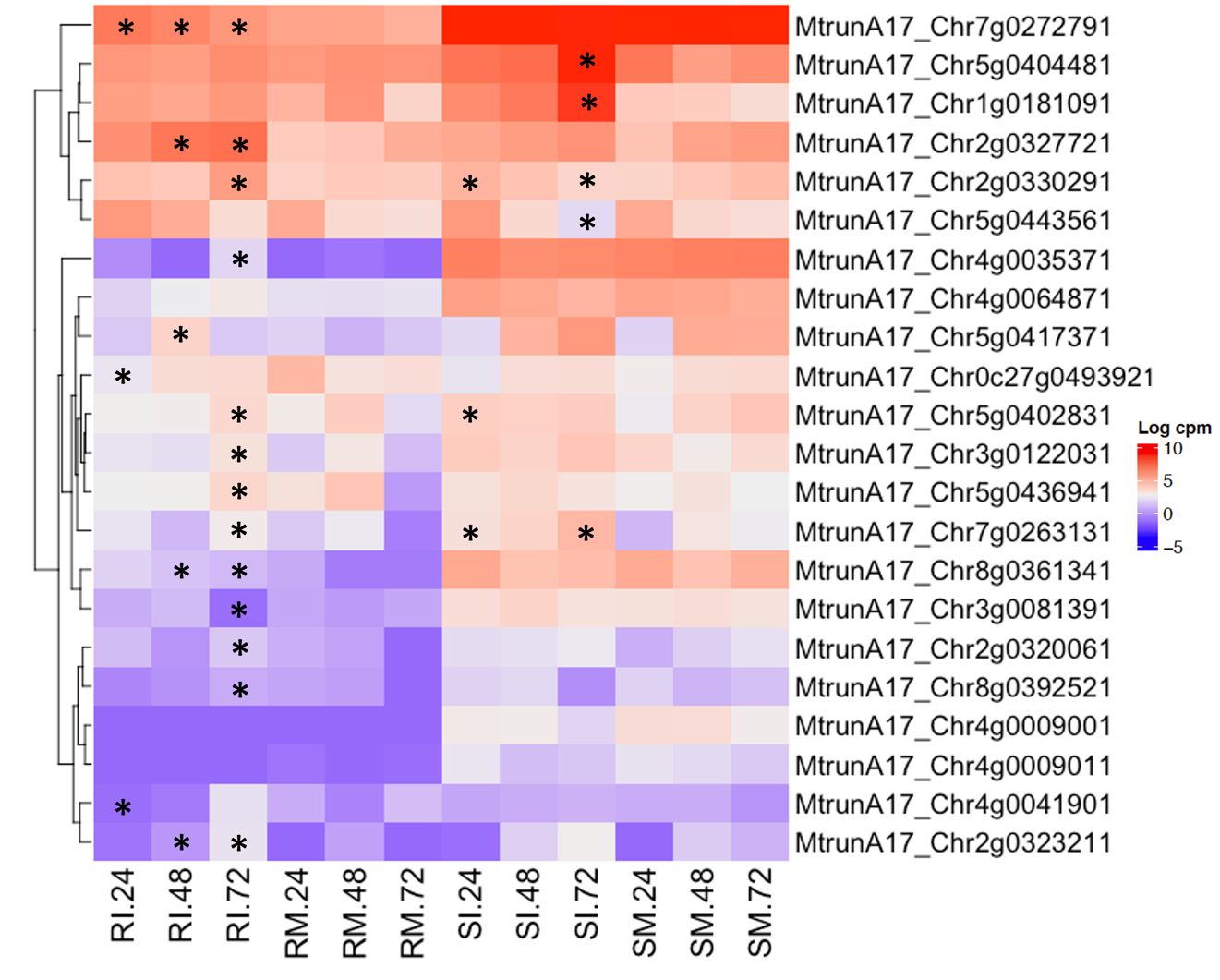


**Figure S8.** Expression profiles of candidate genes for SBS disease resistance. DEGs are marked with an asterisk. Sample ID abbreviations are SM: susceptible mock-inoculated, SI: susceptible inoculated, RM: resistant mock-inoculated, RI: resistant inoculated, followed by hours post inoculation (24, 48, or 72 hpi).


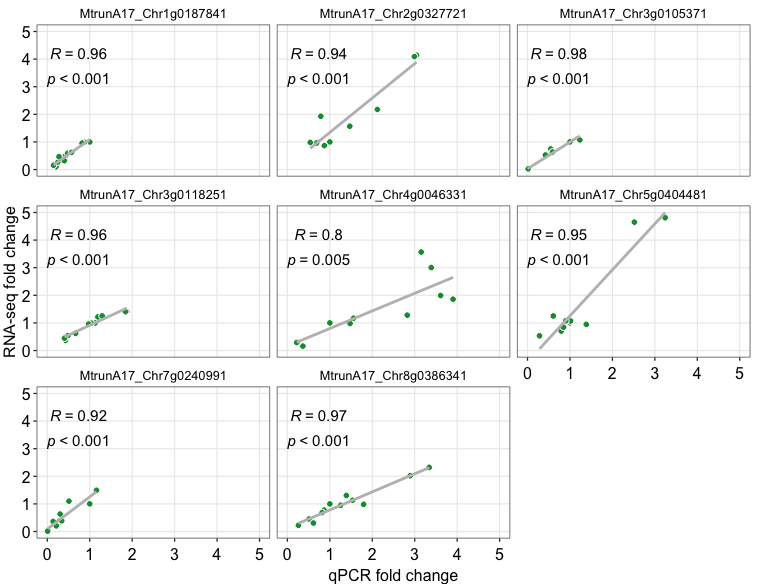


**Figure S9.** Pearson’s correlation of fold change values was performed to compare RNA-seq and qPCR expression data. Genes included were Nepenthesin (MtrunA17_Chr1g0187841), *MtKCS12* (MtrunA17_Chr2g0327721), *MtPP2C* (MtrunA17_Chr3g0105371), *MtEDS1L*-like (MtrunA17_Chr3g0118251), *MtCYP93C19* (MtrunA17_Chr4g0046331), *MtIFR* (MtrunA17_Chr5g0404481), *MtLAC7* (MtrunA17_Chr7g0240991), and *MtP21*-like (MtrunA17_Chr8g0386341).
